# MOGEDN: small-sample cancer subtype classification with encoder–decoder networks for missing-omics recovery and biomarker discovery

**DOI:** 10.1093/bib/bbaf698

**Published:** 2025-12-31

**Authors:** Dingnan Jin, Yutaka Saito

**Affiliations:** Graduate School of Frontier Sciences, The University of Tokyo, 5-1-5 Kashiwanoha, Kashiwa, Chiba 277-0882, Japan; Graduate School of Frontier Sciences, The University of Tokyo, 5-1-5 Kashiwanoha, Kashiwa, Chiba 277-0882, Japan; Artificial Intelligence Research Center, National Institute of Advanced Industrial Science and Technology (AIST), 2-4-7 Aomi, Koto-ku, Tokyo 135-0064, Japan; School of Frontier Engineering, Kitasato University, 1-15-1 Kitazato, Minami-ku, Sagamihara, Kanagawa 252-0373, Japan

**Keywords:** cancer subtype classification, multi-omics integration, missing omics recovery, small-sample learning, biomarker discovery

## Abstract

Effective cancer subtype classification from multi-omics data remains challenging due to incomplete omics data and limited sample sizes. While graph convolutional networks (GCNs) have been used to incorporate inter-sample relationships for enhancing small-sample classification, their performance deteriorates when a certain omics modality is entirely missing. Here, we propose MOGEDN, a novel framework for cancer subtype classification using multi-omics encoder–decoder networks designed to reconstruct the latent features of missing omics data. The reconstructed features are integrated with available omics features to enable robust prediction under small-sample and missing-omics settings. We develop a step-wise algorithm to pretrain our model with diverse cancer types then to finetune for a specific cancer type while incorporating inter-sample and cross-omics dependencies. Evaluated on TCGA cancer datasets including subtypes with fewer than 50 samples, MOGEDEN consistently outperforms state-of-the-art baselines in accuracy and F1 scores. Moreover, MOGEDN’s feature analysis provides two complementary biomarker sets: biomarkers shared across diverse cancer types in the pretraining phase; and biomarkers for a specific cancer type in the finetuning phase, facilitating model interpretability, and biological findings. These results highlight decoder-based imputation as a powerful approach to enhance multi-omics learning, delivering accurate classification, robust few-shot performance, and multi-scale biomarker discovery in incomplete multi-omics cohorts.

## Introduction

Cancer exhibits significant molecular heterogeneity, posing considerable challenges for accurate cancer subtype classification, which is critical for improving diagnosis, prognosis, and personalized treatment [1–5]. Large-scale initiatives such as The Cancer Genome Atlas (TCGA) have comprehensively characterized over 11 000 tumors across 33 cancer types using multi-omics profiling, including mRNA expression, DNA methylation, and microRNA (miRNA) expression [6, 7]. These richly annotated datasets provide a valuable foundation for developing machine learning and deep learning models aimed at tasks, such as cancer subtype classification and biomarker discovery. However, these datasets are often incomplete, with missing omics modalities in a substantial proportion of samples [8].

Recent studies emphasize the importance of developing computational methods that integrate multi-omics data while accounting for incomplete observations [8–10]. Particularly, missing data in real-world clinical settings may arise from technical limitations, cost constraints, or sample quality issues, making robust modeling under the missingness essential [11].

Recent advances in deep learning have seen widespread adoption of graph-based neural network models for cancer subtype classification using multi-omics data. These models leverage graph structures to capture inter-sample topological relationships, enabling improved integration of heterogeneous multi-omics data [12]. Representative architectures include graph convolutional networks (GCNs), graph attention networks, and their variants, which have demonstrated strong performance across diverse biomedical classification tasks [13, 14]. Among these, MOGONET stands out as a pioneering framework that employs GCNs to incorporate a patient similarity graph for each omics modalities, using omics-specific encoders followed by a fused classification layer [15]. This architecture effectively integrates topological structures and omics-specific features to improve classification accuracy. In parallel, attention-based models such as MOADLN have been proposed to explicitly model interactions across omics modalities using self-attention and fusion layers [16], offering alternative strategies for multi-omics integration.

Beyond these frameworks, recent deep learning-based studies on multi-omics cancer analysis can generally be divided into two categories: (i) subtype classification, which assigns samples to predefined molecular subtypes using supervised approaches such as MOGONET and CustOmics [17]; and (ii) subtype identification, which aims to discover novel cancer subtypes in an unsupervised manner, as demonstrated by DILCR [18], DLSF [19], and Subtype-WGME [20]. This study focuses on the former—supervised subtype classification—which plays a crucial role in precision oncology where reliable subtype prediction directly informs diagnosis, prognosis, and treatment stratification.

However, a major limitation of these models lies in their assumption of complete or nearly complete omics data availability during both training and inference. They are not explicitly designed to handle real-world scenarios where entire omics modalities may be missing for subsets of samples. Their performance and applicability degrade significantly when faced with incomplete datasets.

To tackle this issue, methods like CancerSD and CLCLSA have been proposed with mechanisms to impute missing omics features using contrastive learning and feature reconstruction [21, 22]. These approaches work well under partial missingness (i.e. each sample retains some portion of each omics modality) but cannot handle complete missingness where a certain omics modality is entirely missing for a subset of samples. Without available omics-specific features, their decoders lack adequate conditioning information to generate useful features.

Here we propose MOGEDN, a novel framework that couples GCN-based feature extraction with a multi-head decoder architecture designed to explicitly address the challenge of complete omics missingness in both training and inference. We introduce a dedicated decoder module that reconstructs the feature of a missing omics modality from the remaining ones, enabling robust cross-omics feature recovery even under extreme missing conditions. This allows MOGEDN to generate meaningful features even when a certain omics modality is entirely unavailable for a given sample.

Beyond its robustness to omics missingness, MOGEDN demonstrates strong performance on small sample sizes (*n* < 100), a critical setting in rare cancer studies [23–25]. Unlike previous methods that typically rely on large, well-curated datasets, we evaluate our approach on multiple TCGA cancer types with small sample sizes, revealing its generalizability and stability under data scarcity.

We first pretrain the omics-specific GCN encoders and the multi-head decoder on diverse cancer types with a hybrid objective that combines subtype classification with cross-omics reconstruction under random modality masking. We then finetune on each target cancer type, allowing the decoder and classification heads to adapt to tumor-specific distributions while retaining the benefits of the pretrained encoders. This step-wise strategy transfers cross-omics structure learned from data-rich cohorts to scarce regimes, markedly improving stability and generalization in small-sample settings (*n* < 100) and under complete modality missingness (i.e. when an entire omics modality is absent).

Comprehensive evaluation across 17 TCGA cancer types demonstrates that MOGEDN achieves superior accuracy and F1 scores compared with existing baselines under both complete and incomplete multi-omics settings. Additionally, MOGEDN identifies biomarkers that are consistent with known cancer-type-specific and pan-cancer biological signatures, validating the interpretability and biological relevance of the learned features.

## Materials and methods

We developed MOGEDN, a framework for robust cancer-subtype prediction from incomplete multi-omics data. The framework is composed of (i) omics-specific graph convolutional encoders, (ii) a multi-head decoder that reconstructs missing modalities in latent space, and (iii) classifier heads and a view correlation discovery network (VCDN) fusion classifier (Supplementary Fig. S1) for subtype prediction. Training proceeds in three phases (Supplementary Algorithm S1): supervised encoder and classifier pretraining (Phase 1), decoder pretraining (latent reconstruction) (Phase 2), and joint pretraining with decoder reconstruction (Phase 3). For evaluation, pretrained modules were finetuned for each of the external datasets with new classifier heads, and performance was compared under conditions with or without decoder reconstruction (Supplementary Algorithm S2).

### Data collection and preprocessing

We obtained multi-omics data from TCGA, a publicly available resource that provides molecular and clinical profiles across diverse cancer types. For each TCGA cancer type, we downloaded mRNA expression, DNA methylation, and microRNA expression (miRNA) profiles, together with cancer-subtype annotations. To ensure comparability across omics, we only retained samples for which all three modalities (mRNA, DNA methylation, and miRNA) were simultaneously available together with valid subtype labels. This strict intersection guarantees that downstream training and evaluation rely on consistent and matched sample sets across omics. The original dimensionality of each sample is high: approximately (i) mRNA expression: 60 660 genes, (ii) DNA methylation: 485 577 CpG sites, and (iii)miRNA expression: 1881 miRNAs.

To make learning tractable and to focus on biologically informative signals, we adopted a two-step feature selection strategy, distinguishing between pretraining datasets and evaluation datasets.

- Pretraining datasets:
  We extracted both common features and tumor-specific features.
  - Common features were defined as the most variable and frequently measured features across multiple datasets, resulting in 200 features from mRNA, 200 features from DNA methylation, and 100 features from miRNA.
  - Tumor-specific features were further selected within each cancer type, focusing on high-variance, disease-informative features. For each modality, we extracted 800 features from mRNA, 800 features from DNA methylation, and 400 features from miRNA.
  - Combining common and tumor-specific subsets yielded final dimensionalities of 1000 for mRNA, 1000 for DNA methylation, and 500 for miRNA.
    These feature sets were used to train the shared encoders and decoder in Phases 1–3 of pretraining (Supplementary Algorithm S1).
- Evaluation datasets:
  For cancer-type-specific finetuning and testing, we focused only on tumor-specific features, not requiring feature alignment with the pretraining phase. The evaluation feature dimensionalities were set to 1000 for mRNA, 1000 for DNA methylation, and 500 for miRNA.

Finally, all features were normalized within each modality. mRNA and miRNA expression values were log-transformed and standardized, DNA methylation used β-values, and each feature was z-score normalized across samples:


$$ {x}_{ig}^{\mathrm{std}}=\frac{x_{ig}-{\mu}_g}{\sigma_g+\varepsilon } $$

where ${\mu}_g$​ and ${\sigma}_g$​ denote the feature mean and standard deviation, and $\varepsilon$ is a small constant to avoid division by zero.

This preprocessing pipeline ensured that both pretraining and evaluation phases relied on high-quality feature sets, while balancing between cross-dataset common signals and dataset-specific tumor signals.

### Graph construction

For each omics modality, we constructed a sample–sample similarity graph based on cosine distance. Specifically, pairwise cosine distances were computed among samples, and a global distance threshold was chosen such that, on average, each sample retained approximately *k* neighbors (controlled by the parameter $edge\_ per\_ node$). Edges were then weighted by cosine similarity ${s}_{ij}=1-{d}_{ij}$ ​ for pairs within the threshold. We augmented the graph with self-loops, and then row-normalized (L1 normalization across each row). This produced a sparse adjacency suitable for graph convolutional encoders.

During evaluation, we constructed transductive train-test graphs by concatenating training and test samples within a dataset. We then built a single graph on the union using the same global thresholding rule; edges can exist both within and across the train/test partitions, ensuring that test samples were embedded in relation to the training samples without introducing label information.

### Model architecture (Fig. 1 and Supplementary Fig. S1)

#### MOGEDN model consists of the following components

- Encoders ${E}_o$: GCNs mapping features of each omics $o$into latent features ${Z}_o$.
- Decoder $D$: Multi-head network reconstructing the latent of a missing omics from the concatenated features of others (Supplementary Fig. S1).
- Classifier heads ${C}_o$: Single-omics classifiers generating per-modality logits.
- Fusion classifier $C$: A VCDN integrating per-classifier heads outputs for final prediction.

#### Integration among model components

1) Decoder design and integration with encoders.

The decoder is implemented as a single shared multi-head network. It receives the latent representations of the available omics as input and reconstructs the latent representation of the missing omics. The network contains a small fully connected “core” followed by several output heads, each corresponding to one omics type. During the second training stage (decoder pretraining), the model learns to recover the latent representation of a masked omics by minimizing the reconstruction difference between the predicted and true latent features. In the third stage (joint fine-tuning), the encoders, shared decoder, and classifiers are optimized together so that the latent spaces across omics become aligned and complementary. This integration enables the model to better handle missing-omics scenarios during inference.

2) Classifier: single-omics heads and VCDN fusion (Supplementary Fig. S1).

Each omics-specific classifier head predicts subtype logits from its own latent representation, providing an independent view of the same biological sample. However, these logits describe class probabilities within each omics domain and therefore fail to capture higher-order relationships across modalities. To address this, MOGEDN employs a VCDN [26] as a learnable fusion module. In VCDN, the outputs from all classifier heads are first normalized to represent class-wise confidence for each omics. These vectors are then combined to form a joint representation that enumerates all possible class co-occurrences across omics. This tensor encodes how strongly different omics agree or disagree on each subtype prediction. The resulting high-order feature is flattened and passed through a lightweight multilayer perceptron that learns nonlinear dependencies among omics at the class level and produces the final subtype logits. This design allows VCDN to move beyond simple concatenation or averaging, capturing inter-omics complementarity and conflict explicitly in the decision space. During evaluation, the earlier fusion layers are frozen to preserve the learned cross-omics correlations, while only the last linear layer remains trainable to adapt to new data distributions.

### Pretraining framework

#### Phase 1: supervised encoder and classifier pretraining

Encoders and classifiers were trained in a supervised manner using a class-weighted, sample-weighted focal loss (Supplementary Algorithm S1 - Phase 1):


$$ {L}_{\mathrm{cls}}=-\frac{1}{N}{\sum_{i=1}^N}{w}_i{\sum_{c=1}^C}{\mathrm{\alpha}}_c\;{y}_{ic}\;{\left(1-{p}_{ic}\right)}^{\mathrm{\gamma}}\;\log{p}_{ic} $$

where ${p}_{ic}$ is the predicted probability for class $c$, ${y}_{ic}$​ is the one-hot label, ${\alpha}_c$​ are balanced class weights, and ${w}_i$​ are per-sample weights computed to mitigate class imbalance. Early stopping was based on validation F1-macro, and the best checkpoints were saved.

#### Phase 2: decoder pretraining (latent reconstruction)

With encoders frozen, the decoder was trained to reconstruct the latent representation of a masked modality (Supplementary Algorithm S1 - Phase 2). For each dataset and masked ${o}_{\mathrm{mask}}$:


$$ {\hat{Z}}_{o_{\mathrm{mask}}}=D\left(\mathrm{concat}\left({Z}_j\right),j\ne{o}_{\mathrm{mask}}\right) $$

and the reconstruction loss was:


$$ {L}_{\mathrm{recon}}=\mathrm{MSE}\left({\hat{Z}}_{o_{\mathrm{mask}}},{Z}_{o_{\mathrm{mask}}}\right) $$

A similarity score $\mathrm{Sim}=1/\left(1+ MSE\right)$ was monitored per modality, and the average similarity across modalities was used for validation and early stopping.

#### Phase 3: joint pretraining with decoder reconstruction

All modules were jointly trained under simulated missing-modality conditions (Supplementary Algorithm S1 - Phase 3). At each epoch, one modality was randomly masked with probability $P$, and its latent was replaced with the decoder reconstruction.

The overall loss combined classification and reconstruction:


$$ {L}_{\mathrm{total}}={L}_{\mathrm{cls}}+\mathrm{\lambda}\;{L}_{\mathrm{recon}} $$

where $\mathrm{\lambda}$ balances the two terms. Early stopping was based on validation F1-macro.

Evaluation framework

For each external dataset, new single-omics heads and a VCDN fusion classifier were initialized and fine-tuned with pretrained encoders (and optionally the decoder). The evaluation procedure is described in Supplementary Algorithm S2:

- Masked modality: If ${o}_{mask}$was masked, it was either reconstructed using the decoder ($use\_ decoder= True$) or replaced with a zero vector ($use\_ decoder= False$).
- Loss: Weighted cross-entropy was used:
  $$ {L}_{\mathrm{eval}}=\frac{1}{N}{\sum_{i=1}^N}{w}_i\;\left(-\log \left({p}_{i,{y}_i}\right)\right) $$

where weights ${w}_i$​ were derived from balanced class weights.

- Optimization: Adam optimizer with LR scheduling (factor = 0.7, patience = 10).
- Model selection: The best epoch was chosen by a composite score:
  $$ \mathrm{Score}=0.2\times ACC+0.2\times F{1}_{\mathrm{weighted}}+0.6\times F{1}_{\mathrm{macro}} $$

### Evaluation metrics

Model performance was reported using accuracy (ACC), weighted F1-score, and macro F1-score:

- Accuracy (ACC):
  $$ ACC=\frac{Number\ of\ correct\ predictions}{Total\ number\ of\ samples} $$
- Weighted F1-score:
  $$ F{1}_{weighted}=\sum_{c=1}^C\frac{n_c}{N}\bullet \frac{2\bullet Precisio{n}_c\bullet Recal{l}_c}{Precisio{n}_c+ Recal{l}_c} $$

where ${n}_c$ ​ is the number of samples in class $c$.

- Macro F1-score:
  $$ F{1}_{macro}=\frac{1}{C}\sum_{c=1}^C\frac{2\bullet Precisio{n}_c\bullet Recal{l}_c}{Precisio{n}_c+ Recal{l}_c} $$

These metrics ensure a balanced evaluation of both majority and minority subtypes.

### Biomarker identification

We identify discriminative biomarkers at two levels: (i) shared biomarkers learned from the pretraining datasets (common across tumors), and (ii) tumor-specific biomarkers obtained from the fine-tuned model on each evaluation dataset. Both procedures rely on a simple, model-agnostic input × gradient attribution over the single-omics classifier heads operating on encoder latent.

#### Inputs and dimensionalities

Pretraining datasets (used in Supplementary Algorithm S1): from each modality we first selected common features (mRNA 200, DNA 200, and miRNA 100) across datasets, and tumor-specific features (mRNA 800, DNA 800, and miRNA 400) for each dataset, yielding final pretraining input sizes of (1000, 1000, and 500) for mRNA, DNA, and miRNA, respectively.

Evaluation datasets (used in Supplementary Algorithm S2): we used only the tumor-specific features with the same sizes (1000, 1000, and 500).

#### Encoders and heads used for attribution

For each modality *o*∈ {1,2,3} (mRNA, DNA methylation, and miRNA), we used the trained graph encoder ${E}_o$​ (GCN, hidden dims [200,100], latent dim =100) and its single-omics classifier head ${C}_o$​ ​ ($latent\to logits$). During attribution we use an identity adjacency $A=I$​ so that the encoder receives per-sample signals without additional graph effects (consistent with the provided code path).

#### Attribution score

Given a dataset-specific input matrix $ X\in{\mathbb{R}}^{N\times d}$ for one modality, we compute logits ${f}_i={C}_o\kern0em \left({E}_o\kern0em \left({X}_i,I\right)\right)$ for sample ${\it i}$. We form a pseudo-label by the model’s own prediction ${y}_i^{\ast }=\mathrm{arg\,ma}{\mathrm{x}}_{\mathrm{c}}\,{f}_{ic}$ and define the per-sample loss ${\ell}_i= CE\left( fi,{y}_i^{\ast}\right)$. The input × gradient attribution for feature ${\it g}$ at sample ${\it i}$


$$ {a}_{ig}=\left|\;{X}_{ig}\cdotp \frac{\partial{\ell}_i}{\partial{X}_{ig}}\right|. $$

We aggregate over samples to obtain a per-feature importance score


$$ {s}_g=\frac{1}{N}{\sum_{i=1}^N}{a}_{ig}. $$

In the implementation, we backpropagate once per batch with $retain\_ graph= True$ and then take the absolute mean of $X.\mathit{\operatorname{grad}}\odot X$ across samples.

#### Shared biomarkers from pretrained models

Using pretrained encoders${E}_o$ and heads ${C}_o$​ (Supplementary Algorithm S1), we iterate over all pretraining datasets and compute per-cancer scores ${s}_g^{(p)}$ ​ as above. We then average across cancer:


$$ \overline{s_g}=\frac{1}{\mid{\mathcal{P}}_{pre}\mid}\sum_{P\in \mathcal{P}}{s}_g^{(p)}. $$

Because the shared signal is defined over common features, we restrict to the first ${d}_{shared}$​ indices per modality (mRNA 200, DNA 200, and miRNA 100) according to the common-feature lists assembled during pretraining. We finally rank features within this shared subset by $\overline{s_g}$​ and report the top-$k$ (e.g. $k$ =100 in our implementation).

#### Tumor-specific biomarkers from fine-tuned models

For each evaluation dataset $P$ (Supplementary Algorithm S2), we load the fine-tuned encoders and single-omics heads $\left\{{E}_o^{(P)},{C}_o^{(P)}\right\}$ from the best checkpoint selected without masking and without decoder reconstruction. Using the dataset’s training matrix ${X}^{(P)}$ and identity adjacency, we compute ${s}_g^{(p)}$​ over the full tumor-specific feature sets (mRNA 1000, DNA 1000, and miRNA 500) and rank within the dataset:


$$ {\mathrm{TopK}}^{\left(P,o\right)}=\underset{g\in{\mathcal{F}}^{(o)}\ }{\mathrm{argsort}}\left({s}_g^{(P)}\right)\left[\;1:k\;\right], $$

with $k=300$ by default.

## Results

### Model overview and workflow

We propose a graph-based deep learning framework for robust multi-omics cancer subtype classification, explicitly designed to handle missing omics modalities. The model integrates inter-sample topological structures via GCNs and employs an omics-specific multi-head decoder to reconstruct missing latent features. This design enables the model to perform accurate predictions even under severe omics-level incompleteness and small-sample settings.

**Figure 1:**
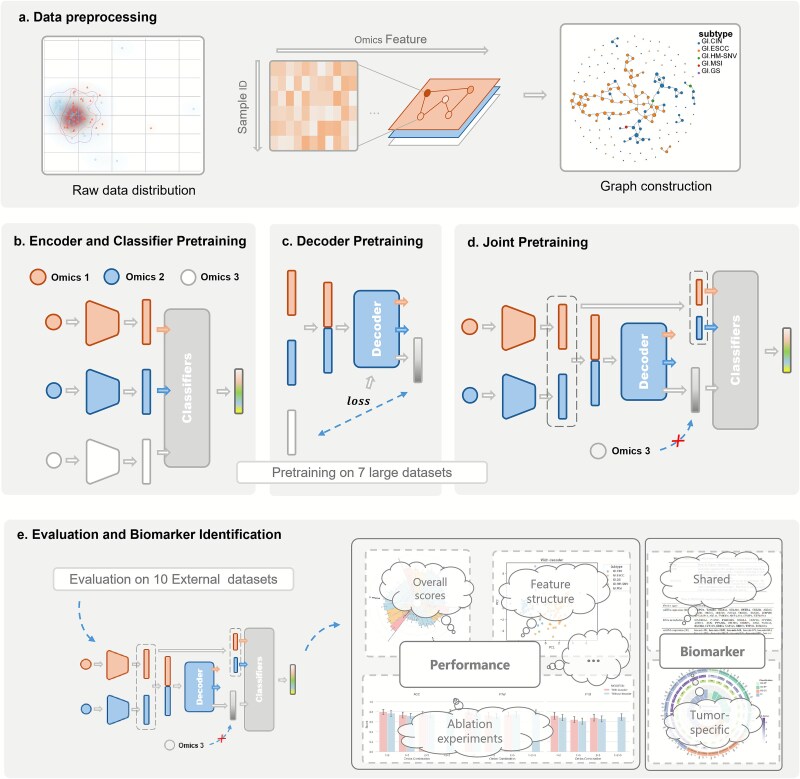
The overview of MOGEDN. (a) Multi-omics data undergo dimensionality reduction and graph building to encode inter-sample relationships. (b) Pretraining phase 1: supervised multi-task training learns omics-specific encoders and classifiers (Supplementary Fig. S1). (c) Pretraining phase 2: decoder is trained to reconstruct the latent feature of a randomly masked omics view, enabling cross-modal inference. (d) Pretraining phase 3: the pretrained encoders are further trained by the classification task in missing omics settings, using both classification and reconstruction loss, then the pretraining phases 1–3 are performed across seven datasets. (e) Final evaluation is performed with the finetuning for each of 10 external datasets, with biological interpretation and biomarker identification via saliency analysis and functional enrichment analysis.

As illustrated in Fig. 1, the workflow consists of five key components: data preprocessing (Fig. 1a), three-phase model pretraining (Fig. 1b–d), and final evaluation and biomarker discovery (Fig. 1e).

#### Data preprocessing

To construct a reliable and biologically meaningful pretraining set, we selected seven large-scale TCGA cancer datasets with complete mRNA, DNA methylation, and miRNA data (Table 1). Each of three omics types independently underwent dimensionality reduction to fixed-length vectors of [1000, 1000, 500], using a two-step procedure: (i) removal of zero-variance features, followed by (ii) feature ranking by variance (Fig. 1a). Shared informative features across datasets are retained to obtain [200, 200, 100] cancer-type common dimensions. In addition, each dataset contributes to unique, high-variance cancer-type-specific features, expanding to [800, 800, 400] dimensions per omics.

**Table 1:** Pretraining TCGA datasets

Dataset	Cancer type	Cancer subtypes	No. training samples	No. validation samples
TCGA-BRCA	Breast Invasive Carcinoma	BRCA.Normal BRCA.LumA BRCA.Her2 BRCA.LumB BRCA.Basal	532	229
TCGA-PCPG	Pheochromocytoma and Paraganglioma	PCPG.Kinase signaling PCPG.NA PCPG.Wnt-altered PCPG.Pseudohypoxia PCPG.Cortical admixture	118	52
TCGA-LIHC	Liver Hepatocellular Carcinoma	LIHC.iCluster:1 LIHC.iCluster:2 LIHC.iCluster:3 LIHC.NA	130	57
TCGA-COAD	Colon Adenocarcinoma	GI.CIN GI.MSI GI.GS GI.HM-SNV	182	78
TCGA-UCEC	Uterine Corpus Endometrial Carcinoma	UCEC.CN_HIGH UCEC.CN_LOW UCEC.POLE UCEC.NA UCEC.MSI_H	289	125
TCGA-STAD	Stomach Adenocarcinoma	STAD.EBV STAD.MSI STAD.Genome Stable STAD.Chromosomal Instability STAD.NA	238	103
TCGA-HNSC	Head and Neck Squamous Cell Carcinoma	HNSC.Atypical HNSC.Basal HNSC.Classical HNSC.Mesenchymal	193	84

We built per-omics sample graphs based on pairwise cosine distances (1-cosine similarity), selecting a global distance threshold tuned to yield approximately *k* edges per node (Fig. 1a). We augmented the graph with self-loops and applied row-wise L1 normalization so that each row (outgoing weights) sums to one.

For evaluation, we used 10 external TCGA datasets of medium-to-small sizes (Table 2). There was no overlap of cancer types with the pretraining datasets (Table 1). For each dataset, variance-based feature selection was applied, reducing each omics to [1000, 1000, 500] dimensions without enforcing cross-dataset feature alignment.

**Table 2:** Evaluation TCGA datasets

Dataset	Cancer type	Cancer subtypes	No. training samples	No. test samples
TCGA-KIRC	Kidney renal clear cell carcinoma	KIRC.1 KIRC.2 KIRC.3 KIRC.4 KIRC.NA	175	75
TCGA-READ	Rectum adenocarcinoma	GI.CIN GI.GS GI.HM-SNV GI.MSI	60	26
TCGA-ESCA	Esophageal carcinoma	GI.CIN GI.HM-SNV GI.ESCC GI.GS GI.MSI	111	48
TCGA-KICH	Kidney chromophobe	KICH.Eosin.0 KICH.Eosin.1	45	20
TCGA-UCS	Uterine carcinosarcoma	UCS.1 UCS.2	39	18
TCGA-KIRP	Kidney renal papillary cell carcinoma	KIRP.C2a KIRP.C1 KIRP.C2c - CIMP KIRP.C2b	102	45
TCGA-ACC	Adrenocortical carcinoma	ACC.CIMP-high ACC.CIMP-low ACC.CIMP-intermediate	54	24
TCGA-SKCM	Skin cutaneous melanoma	SKCM.BRAF SKCM.NF1 SKCM.RAS SKCM.Triple Wild-Type SKCM.NA	45	18
TCGA-BLCA	Bladder urothelial carcinoma	BLCA.Luminal BLCA.Basal BLCA.Neuroendocrine BLCA.Stroma-rich	88	39
TCGA-LUSC	Lung squamous cell carcinoma	LUSC.Luminal LUSC.Basal LUSC.Classical LUSC.Secretory	51	22

#### Pretraining on seven large datasets

##### Phase 1: supervised encoder and classifier pretraining

In the first phase, omics-specific encoders and classifiers are trained using samples with complete modalities (Fig. 1b). Each encoder maps omics data to a feature, while a corresponding classifier predicts subtype labels. The predicted labels from classifiers are further combined across omics and used as features for a fusion classifier (Supplementary Fig. S1), which captures cross-omics dependencies and enhances subtype discrimination. This phase ensures that each encoder learns informative, omics-specific features for subtype classification.

##### Phase 2: decoder pretraining (latent reconstruction)

To learn to impute missing modalities, we randomly mask one omics type per training sample and train a multi-head decoder to reconstruct the missing feature using the remaining omics views (Fig. 1c). The decoder is trained with a reconstruction loss that minimizes the discrepancy between the predicted and ground-truth latent features. Importantly, this phase is unsupervised and is performed after freezing the encoders from Phase 1. It allows the decoder to learn cross-omics dependencies without relying on subtype label information.

##### Phase 3: joint pretraining and decoder reconstruction

In the final phase of pretraining (Fig. 1d), the decoder is frozen to prevent overfitting, while the encoders and classifiers’ weights are further adjusted using a hybrid input: observed features for available modalities and decoder-reconstructed latent features for missing ones. This joint adaptation process simulates realistic clinical scenarios where certain omics may be absent. By exposing the classifier to mixed real and reconstructed inputs, the model learns to maintain predictive robustness under variable omics configurations.

A high-level pseudocode outlining the pretraining procedure is presented in Supplementary Algorithm S1.

#### Evaluation and biomarker identification

The pretrained model was finetuned and evaluated on each of the 10 external TCGA datasets under both complete and missing-omics conditions (Fig. 1e). During this stage, the decoder remains frozen, and classifier weights are partially frozen to prevent overfitting on small-sample datasets. We assessed model performance using accuracy, F1-weighted, and F1-macro scores. In addition to subtype classification, the model enables biomarker discovery by analyzing feature attributions. The pretrained model identifies biomarkers shared across cancer types used in the pretraining dataset (Table 1), while the fine-tuned models uncover biomarkers for a specific cancer type for each of the evaluation dataset (Table 2).

#### Performance on complete multi-omics datasets

**Figure 2:**
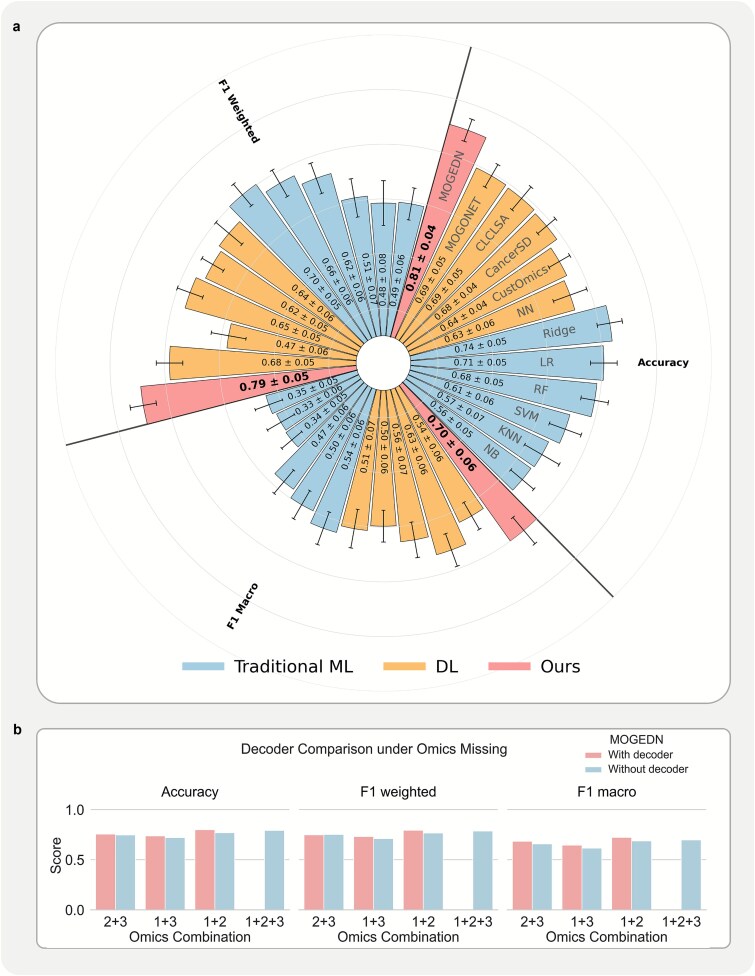
Performance on 10 external TCGA datasets. (a) Average classification performance across all datasets using complete multi-omics input, where MOGEDN is compared with traditional machine learning (ML) models and the existing methods based on deep learning (DL). NN, neural network; ridge, ridge regression; LR, logistic regression; RF, random forest; SVM, support vector machine; kNN, *k*-nearest neighbors; NB, naive bayes. (b) Average performance of MOGEDN with and without decoder across all missing-omics scenarios. Omics1, mRNA expression; Omics2, DNA methylation; Omics3, miRNA expression.

To assess the baseline effectiveness of MOGEDN, we first evaluated its performance on ten external TCGA datasets with complete multi-omics profiles (mRNA, DNA methylation, and miRNA). As shown in Fig. 2a, our method consistently outperformed both traditional machine learning classifiers and existing deep learning baselines across multiple datasets.

F1-weighted, F1-macro, and accuracy metrics for each dataset are reported in Supplementary Tables S1–S10, highlighting the superiority of our fusion-based classification strategy. Notably, MOGEDN demonstrated significant advantages in datasets with limited sample sizes, such as TCGA-READ, TCGA-KICH, TCGA-ACC, TCGA-UCS, TCGA-SKCM, TCGA-BLCA, and TCGA-LUSC (number of training samples <100) confirming its robustness under data scarcity.

#### Decoder-based improvement under missing omics data

We next investigated MOGEDN’s ability to handle omics-level missingness by evaluating it under single-omics missing settings (e.g. missing mRNA, missing DNA methylation, or missing miRNA). In this setting, the decoder was used to reconstruct the features of the missing omics, which were then fused with available omics features for subtype prediction.

As shown in Supplementary Fig. S2, across all TCGA evaluation datasets, the model incorporating the decoder achieved improvements in at least two out of three metrics (ACC, F1-weighted, and F1-macro) compared with the ablated variant without the decoder. This demonstrates that decoder-based latent reconstruction consistently enhances performance across diverse omics configurations.

To provide a clearer summary of this effect, we further calculated average performance scores across datasets under each omics combination. As illustrated in Fig. 2b, the decoder-equipped model achieved substantial gains across all three metrics under most combinations. Notably, in the mRNA and DNA methylation omics setting (i.e. miRNA features were imputed by the decoder), the model’s performance was comparable to or even exceeded that under the complete omics setting (mRNA, DNA methylation, and miRNA). This observation suggests that the pretrained decoder has the capacity to infer meaningful features that extend beyond the directly observed information, highlighting the model’s potential to generalize beyond the scope of the training data.

**Figure 3:**
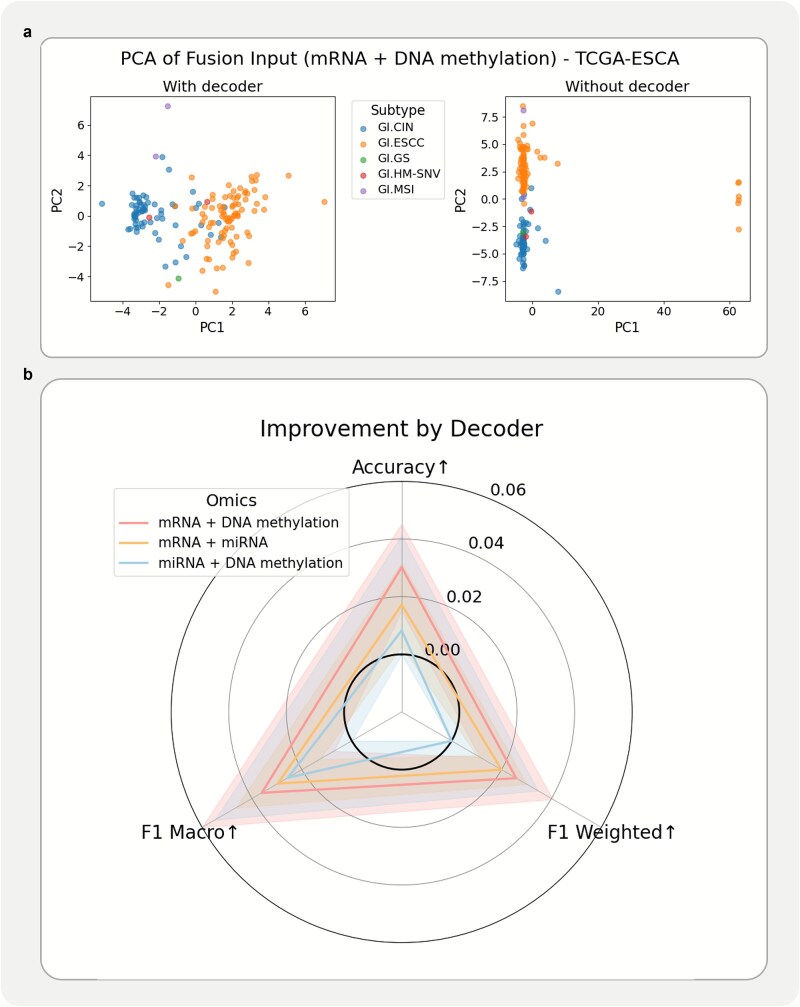
Improvement by the decoder’s reconstruction of missing omics data. (a) PCA visualization of features extracted with and without the decoder, highlighting differences in feature structure. (b) Radar plot showing the decoder’s improvement for three omics combinations, where solid lines correspond to the mean performance gain in accuracy, F1 weighted, and F1 macro; shaded regions denote the standard error of the mean.

To further quantify the decoder’s contribution, we visualized the fused latent feature space under omics-missing conditions, comparing models with and without decoder reconstruction. As shown in Fig. 3a, the incorporation of the decoder leads to a clearly separable feature distribution, in contrast to the entangled features observed without the decoder. This result confirms that the observed performance gain is not merely due to random chances under limited data but reflects meaningful latent recovery driven by the pretrained decoder.

Moreover, the aggregated comparison across all omics-missing scenarios (Fig. 3b) indicated that miRNA reconstruction yields the greatest benefit, followed by mRNA and DNA methylation. This trend may be due to that miRNA features are lower in dimension, making them more susceptible to loss but also more informative when successfully recovered. Together, these findings validate the decoder’s role in enhancing latent structure quality and support its effectiveness in mitigating missing-omics effects.

**Figure 4:**
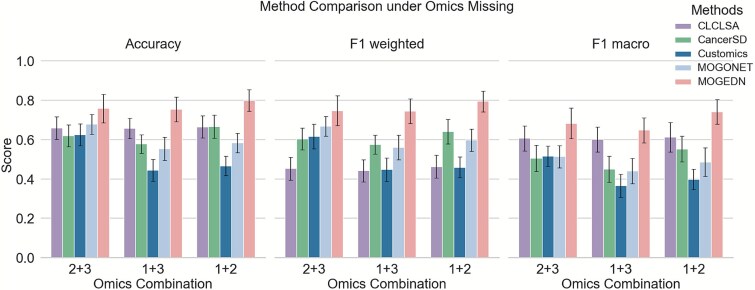
Comparison of representative deep learning models under omics missing, where average classification performance (ACC, F1-weighted, and F1-macro) of five deep-learning-based multi-omics integration models across 10 evaluation TCGA datasets under different missing omics scenarios (Bars denote mean ± SE across datasets). Omics1, mRNA expression; Omics2, DNA methylation; Omics3, miRNA expression.

To benchmark MOGEDN against other deep learning-based integration frameworks, we compared it with four representative models—CLCLSA, CancerSD, CustOmics, and MOGONET—under the same omics-missing settings (Fig. 4). MOGEDN achieved the best overall performance in all three evaluation metrics (Accuracy, F1-weighted, and F1-macro) across every omics combination, indicating its superior capability to maintain predictive accuracy even when one omics modality is absent. While other deep learning models leverage shared latent spaces or attention mechanisms for multi-omics fusion, their performance decreased markedly as data incompleteness increased. Although CancerSD and CLCLSA were explicitly designed to handle partially missing omics data, their performance remained suboptimal when an entire omics modality was missing, suggesting that their fusion strategies are less effective in fully missing-omics scenarios. In contrast, MOGEDN’s decoder enables effective imputation of the missing-omics representations, yielding more balanced and resilient performance across diverse missing-data configurations.

To test our model’s performance not only in small-sample setting but also large-sample settings, we further evaluated MOGEDN on the TCGA-BRCA (Breast Invasive Carcinoma) dataset, which represents one of the largest TCGA projects with over 700 samples. We used six pretraining datasets excluding BRCA for pretraining, then fine-tuned on BRCA, and report the results in Supplementary Table S11. Our method achieved top two performance across all three metrics (Accuracy, F1-weighted, and F1-macro) compared with existing baselines, demonstrating that the proposed framework retains strong efficacy in the large-sample cancer dataset.

#### Biomarker identification

To elucidate the shared molecular mechanisms across multiple cancer types, we applied the pretrained model (Fig. 1b–d) to extract informative features from three omics modalities (mRNA expression, DNA methylation, and miRNA expression), yielding 100, 100, and 50 top-ranked features, respectively. Among these, genes mapped from the selected mRNA and DNA methylation features were subjected to functional enrichment analysis using g: Profiler [27]. As summarized in Table 3, the top enriched Gene Ontology (GO) terms were associated with chromatin remodeling (e.g. “pericentric heterochromatin” and “CHRAC”), protease complexes involved in metastasis (e.g. “serine-type endopeptidase complex”), and membrane-associated neuronal structures increasingly implicated in tumor progression [28–31].

**Table 3:** Top enriched GO and TF terms identified from shared features

Term name	Term ID	Source	Adj. *P*-value	Biological relevance
regulation of somitogenesis	GO:0014807	GO:BP	.0049	Developmental signaling
neuron projection membrane	GO:0032589	GO:CC	.0051	Neural structure
pericentric heterochromatin	GO:0005721	GO:CC	.0061	Chromatin condensation
serine-type endopeptidase complex	GO:1905370	GO:CC	.0330	ECM degradation
CHRAC	GO:0008623	GO:CC	.0499	Chromatin remodeling
E2F-1: Elk-1	TF:M08205_1	TF	.0004	Cell cycle activation
E2F-3	TF:M02089	TF	.0008	G1/S transition
ETF	TF:M00695	TF	.0010	Unknown regulator
RNF96	TF:M01199	TF	.0011	Transcriptional repression
E2F-4	TF:M09894	TF	.0029	Cell cycle repression

In parallel, transcription factor (TF) motif enrichment revealed strong signals from the E2F family (E2F-1, E2F-3, and E2F-4) and ELK-1, which are key regulators of cell cycle entry and proliferative signaling. The consistent appearance of these TFs suggests that core transcriptional dysregulation underlies shared oncogenic programs across diverse tumor types. Together, these results underscore the role of epigenetic alterations and transcriptional misregulation as common alterations in cancer.

Among the shared features extracted from multi-omics data, *EZH2* and *NOTCH1* were identified as high-confidence pan-cancer driver genes, supported by large-scale cancer genomics studies, such as TCGA PanCancer Atlas [6] and COSMIC [32] (Table 4). EZH2 is a histone methyltransferase and core member of the PRC2 complex, which promotes tumor progression via widespread epigenetic silencing of tumor suppressor genes [33]. NOTCH1 is a key component of the Notch signaling pathway, which has been shown to act as either an oncogene or a tumor suppressor depending on tissue context, and is recurrently altered in hematologic and epithelial malignancies [34].

**Table 4:** High-confidence pan-cancer driver genes identified in shared features

Gene	Role in cancer research
*EZH2*	Epigenetic regulator that mediates histone H3K27 trimethylation; recurrently overexpressed or mutated across cancers such as prostate, breast, and lymphoma. Drives oncogenesis by silencing tumor suppressor genes.
*NOTCH1*	Transmembrane receptor involved in cell fate decisions. Frequently mutated in T-cell acute lymphoblastic leukemia, breast cancer, and squamous carcinomas. Functions as both oncogene and tumor suppressor depending on context.

To characterize cancer-specific molecular signatures across distinct tumor types, we performed the feature analysis with our finetuned model for each of the 10 external datasets. From each dataset, we retained 20, 20, and 10 top-ranked biomarkers from mRNA expression, DNA-methylation and miRNA data, respectively. We specifically highlight results for TCGA-ACC (adrenocortical carcinoma), TCGA-BLCA (bladder urothelial carcinoma), and TCGA-ESCA (esophageal carcinoma) because these cohorts span different training sample sizes within our evaluation set (~50, 80, and 110 samples, respectively; see Table 2). This selection thus provides representative cases at small, medium, and larger scales to demonstrate the robustness of our biomarker identification method. Representative sets for these three cancers are listed in Tables 5–7, while full results for the other datasets are provided in Supplementary Tables S12–S18.

**Table 5:** TCGA-ACC associated biomarkers from multi-omics data

Omics type	Biomarkers
mRNA expression (20)	**TRPC5**, TAS2R8, SEMG2, OR7A10, **SSTR4**, CBLN2, ARL5C, ASB10, OR5C1, HS3ST6, PDCL2, CHRM1, TAS2R1, **HSPB2**, RPL7L1P11, ARL14, TMIGD1, NCF4-AS1, CIMIP4, KRT18P14
DNA methylation (20)	DNASE1L1, PTPRF, **PRDM16**, MMEL1, TFAP2E, PPPDE1, AHDC1, **ALK, PPARG**, CELSR3, SH3BP5, ARSJ, FAM13A, HMGB2, CYP4V2, EREG, NAP1L5, HERC3, TNPO1, FAM105A
miRNA expression (10)	hsa-mir-496, **hsa-mir-1249**, **hsa-mir-511**, hsa-mir-671, hsa-mir-3613, hsa-mir-1294, **hsa-mir-101-2**, hsa-mir-373, **hsa-mir-107**, hsa-mir-125b-1

Note: Biomarkers in bold have literature evidence for association with ACC or other cancers.

**Table 6:** TCGA-BLCA associated biomarkers from multi-omics data

Omics type	Biomarkers
mRNA expression (20)	SPO11, PHOX2B, ZSCAN10, CDKN2AIPNLP2, GPR119, **LCN1**, SUN5, **SPACA5**, TERF1P1, **GPRC6A**, ENSAP3, EEF1A1P27, GARIN6, OR6K4P, OR1G1, SPDYE4, PGPEP1L, RPS4XP21, GTF2IP3, LINC01164
DNA methylation (20)	**CALN1**, **PGK1**, SYT6, MAP7D1, C1orf25, C1orf26, LOC284632, CCDC17, LOC388588, TNFRSF25, RFX5, **PINK1**, SLC44A3, NPHP4, PASK, SNRPG, SAP130, CDC25A, ZFYVE20, FRAS1
miRNA expression (10)	**hsa-miR-21**, **hsa-let-7b**, **hsa-miR-6716**, hsa-miR-365a, hsa-miR-130a, hsa-miR-1237, hsa-miR-4674, hsa-miR-1537, hsa-miR-7974, hsa-miR-3909

Note: Biomarkers in bold have literature evidence for association with BLCA or other cancers.

**Table 7:** TCGA-ESCA associated biomarkers from multi-omics data

Omics type	Biomarkers
mRNA expression (20)	**MS4A5**, PDILT, OR10H3, OR1A2, CBLL2, OR7A15P, OR2M1P, OR2G2, FAM187B, VN1R3, OR56B1, SSX7, **GFRAL**, VN1R2, KRTAP4–3, OR5AC2, OR7A11P, RNA5SP426, SPANXN1, RNU6-166P
DNA methylation (20)	AVPR2, RBM10, NDUFB11, BRCC3, **MTCP1**, MTCP1NB, IDH3G, **SSR4**, MAGT1, **KCNAB2**, C1orf122, YRDC, VPS13D, NMNAT2, PPIE, COBLL1, C2orf42, ATP6V1A, NAA50, LOC255025
miRNA expression (10)	hsa-mir-4474, hsa-mir-643, hsa-mir-6892, hsa-mir-3605, **hsa-mir-92a-1**, hsa-mir-6810, hsa-mir-550a-2, **hsa-mir-939**, hsa-mir-3680-2, **hsa-mir-484**

Note: Biomarkers in bold have literature evidence for association with ESCA or other cancers.

Among the TCGA-ACC biomarkers (Table 5), TRPC5, SSTR4, and HSPB2 are linked to adrenal biology or ACC progression. TRPC5 is highly expressed in adrenal chromaffin cells and modulates Ca^2+^-dependent catecholamine release, a pathway frequently altered in adrenocortical tumors [35]. SSTR4, one of the somatostatin-receptor subtypes, is detectable in ACC tissues and has been explored as a target for somatostatin-analog therapy [36]. HSPB2, a small heat-shock protein, is a prognostic marker in kidney renal papillary cell carcinoma and may support stress tolerance of tumor cells [37]. From the DNA-methylation layer, PRDM16 (epigenetically silenced and associated with poor outcome) [38], ALK (previously indicated by altered gene expression in TCGA data and associated with survival in ACC patients) [39], and PPARG (whose agonists suppress ACC-cell proliferation *in vitro*) [40] emerged as top candidates.

Among the 10 miRNA biomarkers retained by the model, four show reproducible evidence of involvement in ACC. miR-107 is consistently over-expressed in ACC tissue and serum compared with adrenocortical adenoma (ACA) and has been proposed as part of a circulating-miRNA panel for differentiating malignant from benign lesions [41]. Extracellular-vesicle profiling further identified miR-101-2 as a discriminator between ACC and ACA, pointing to its utility as a minimally invasive biomarker [42]. Large-scale sequencing of ACC samples revealed significant deregulation of miR-511, which distinguished malignant from nonmalignant adrenal cortex with false-discovery rates <0.05 [43]. Finally, database and mouse-model evidence indicate that miR-1249 is dysregulated in ACC and related adrenal tumors, supporting its candidacy as a cancer-associated miRNA [44]. These converging data underline the biological relevance of the model-selected miRNAs and support their further exploration as ACC-specific diagnostic or prognostic markers.

To probe mechanism, we performed TF-motif enrichment on ACC DNA methylation biomarkers, which highlighted binding motifs for ZF5, E2F-1, HES-7, HA95, and ETF (Table 8). In parallel, GO enrichment revealed significant associations with multicellular organism development, nervous system development, protein binding, cytoplasm, cytosol, and multiple projection-related terms (neuron projection, cell projection, and plasma-membrane-bounded projection) (Table 8). These results indicate contributions from developmental signaling, cytoskeletal remodeling, and cytoplasmic processes.

**Table 8:** Top enriched GO and TF terms identified from TCGA-ACC features

Term name	Term ID	Source	Adj. *P*-value	Biological relevance
multicellular organism development	GO:0007275	GO:BP	1.30e-5	Developmental signaling
protein binding	GO:0005515	GO:MF	4.89e-4	General molecular function
nervous system development	GO:0007399	GO:BP	7.44e-4	Developmental signaling
neuron projection	GO:0043005	GO:CC	4.64e-2	Neural/cytoskeletal projection
Cytoplasm	GO:0005737	GO:CC	2.04e-3	Cytoplasmic localization
cell projection	GO:0042995	GO:CC	2.54e-2	Cellular projection
plasma membrane bounded cell projection	GO:0120025	GO:CC	3.81e-2	Cytoskeletal/projection remodeling
Cytosol	GO:0005829	GO:CC	1.30e-2	Cytoplasmic localization
ZF5	TF:M00716	TF	2.66e-8	Transcriptional regulation
E2F-1	TF:M09892	TF	1.51e-7	Cell cycle activation
HES-7	TF:M08525_1	TF	1.26e-7	Notch signaling regulator
HA95	TF:M13127_1	TF	2.33e-7	Chromatin regulation
ETF	TF:M00695	TF	3.72e-7	Transcriptional regulation

Figure S3 provides a global visualization of all significantly enriched GO and TF terms identified from the enrichment analysis, whereas Table 8 summarizes and interprets the most representative terms that are biologically relevant to ACC.

Of note, E2F-1 cooperates with EZH2 to drive an aggressive ACC transcriptional program and is a recognized predictor of poor prognosis [45]; thus, the enrichment of its binding motif underscores a central role of cell-cycle dysregulation in ACC, while enriched GO terms point to parallel involvement of developmental and structural pathways in tumor progression.

As for the TCGA-BLCA biomarkers (Table 6), among the DNA-methylation features, CALN1 hypomethylation has been proposed as a urinary diagnostic marker that stratifies high-risk, nonmuscle-invasive bladder cancer patients and improves recurrence prediction models [46]. The glycolytic enzyme PGK1 is frequently up-regulated and promotes proliferation, invasion, and platinum resistance in BLCA, and its high expression independently predicts poor overall survival [47]. At the post-transcriptional level, miR-21 is one of the best-validated oncomiRs in bladder cancer; elevated miR-21-5p in tumor tissue or extracellular vesicles correlates with higher stage, early recurrence, and reduced cancer-free survival [48].

Within the TCGA-ESCA biomarker (Table 7), IDH3G, one of the γ-subunits of mitochondrial isocitrate dehydrogenase, shows marked transcriptional up-regulation in TCGA and independent cohorts; its high expression correlates with advanced stage and inferior overall survival [49]. At the miRNA layer, miR-939-3p is elevated in plasma and tumor tissue of ESCC (esophageal squamous cell carcinoma) patients and drives epithelial–mesenchymal transition and metastatic spread [50], underscoring the metabolic and invasive re-programming that characterizes aggressive esophageal cancer.

A full list of biomarkers for the remaining cancer types, including TCGA-KIRC, TCGA-READ, TCGA-KICH, TCGA-UCS, TCGA-KIRP, TCGA-SKCM, and TCGA-LUSC, can be found in Supplementary Tables S11–S17.

## Discussion and Conclusion

### Leveraging incomplete, labeled omics for precision oncology

Recent sequencing initiatives such as TCGA have transformed multi-omics from a purely exploratory resource into a set of increasingly well-annotated, cancer-centered datasets. Yet whole-genome methylation or proteomic assays remain far costlier than bulk RNA-seq; consequently, many clinical cohorts provide at most two omics layers and often contain fewer than 200 patients. Existing integrative models either assume complete data or collapse when an entire omics is absent. To bridge this gap, we present MOGEDN, an encoder–decoder framework that regards each omics type as a view and can infer the feature of a missing view during subtype prediction. The architecture is trained through three pretraining phases that successively establish omics-specific feature, cross-omics reconstruction, and noise-robust classification, after which a lightweight finetuning step adapts the frozen backbone to each new cohort.

### Three-phase pretraining mitigates small-sample over-fitting

Phase 1 trains an encoder and multiple omics-specific classifier heads on every labeled TCGA dataset simultaneously, yielding a pan-cancer prior. Phase 2 freezes the encoder and optimizes a decoder to reconstruct the latent features of a randomly masked omics view; this teaches the network to model inter-omics dependency explicitly rather than rely on imputation at the feature level. Phase 3 re-introduces supervised learning while keeping the decoder active: at every iteration, one omics is masked, replaced by its reconstructed latent features, and the joint loss balances classification with reconstruction. When we subsequently fine-tune on a target cancer dataset, we keep most encoder layers frozen and expose the model to the true missing-omics pattern of that dataset. This curriculum allows the network to generalize to datasets that are both small and modal-sparse, yielding consistently higher accuracy and F1 scores than conventional MLPs, ridge-regression ensembles, and the earlier MOGONET architecture.

### Biological insight and extensibility

Gradient-based saliency on the fine-tuned models recovers biologically plausible markers for each cancer type (Tables 5–7 and Supplementary Tables S12–S18). Because the encoder treats each omics block as an independent graph, adding further data types such as copy number, proteome, or even histology features, requires only an additional view-specific subnetwork and minimal re-training. Importantly, since the encoder is trained across diverse cancers in a unified feature, attribution analysis also reveals shared biomarkers that consistently receive high importance scores across multiple cancer types (Tables 3 and 4), highlighting the potential of the model not only to adapt to individual cancer types, but also to discover pan-cancer molecular features that may inform broad therapeutic strategies or serve as cross-cancer diagnostic markers. MOGEDN provides a generic, extensible, and biologically informed solution for supervised multi-omics analysis in the realistic setting of limited sample size and incomplete omics coverage.

**Key Points**

- We introduce MOGEDN, an encoder–decoder framework that reconstructs missing omics features to enable robust multi-omics cancer subtype classification under incomplete data settings.
- By combining graph convolutional encoders with a multi-head decoder, MOGEDN achieves superior accuracy and F1 scores compared to state-of-the-art baselines, even for subtypes with fewer than 50 samples.
- The step-wise pretraining and finetuning strategy transfers knowledge from data-rich cohorts to rare cancer datasets, enhancing stability under both small-sample and missing-omics conditions.
- Decoder-based reconstruction not only improves prediction but also enables biologically meaningful biomarker discovery at both pan-cancer and cancer-specific levels.
- This framework provides a generalizable and extensible solution for precision oncology where limited sample size and incomplete omics data remain major challenges.

## Supplementary Material

MOGEDN_Suppl_251209_bbaf698

## Data Availability

MOGEDN software is available at https://github.com/KelvinJin08/MOGEDN.
