# Supplementary material for: MOGEDN: small-sample cancer subtype classification with encoder–decoder networks for missing-omics recovery and biomarker discovery
Source: Brief Bioinform. 2025 Dec 31;26(6):bbaf698. doi: 10.1093/bib/bbaf698 (PMC12753307; doi:10.1093/bib/bbaf698)
Supplement: MOGEDN_Suppl_251209_bbaf698 [file mogedn_suppl_251209_bbaf698.pdf]

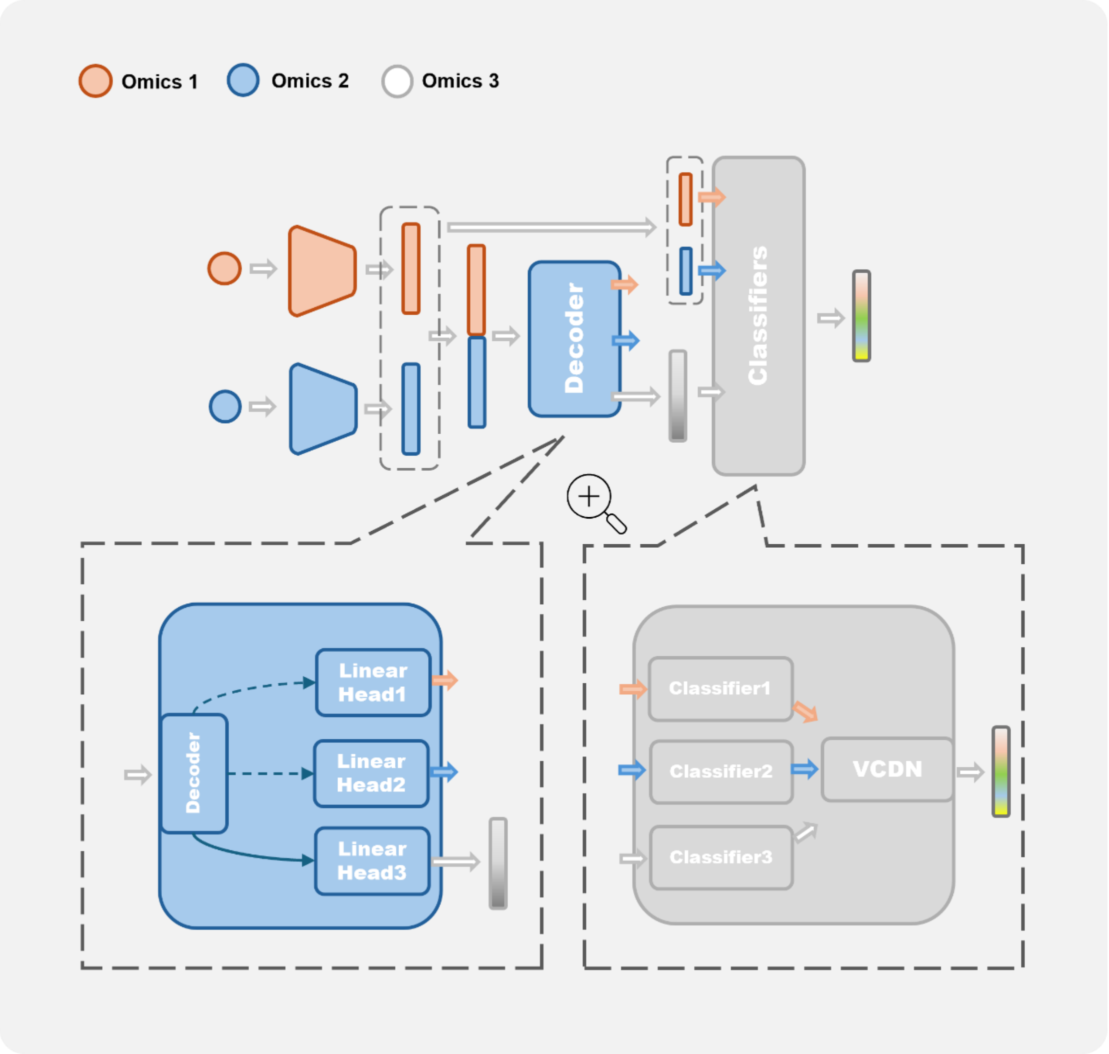

Figure S1 Internal architecture of the decoder and classifier.

Multi-head decoder reconstructing the latent of a missing omics from the concatenated features of others. Classifiers include Single-omics classifiers and a fusion classifier View Correlation Discovery Network (VCDN).

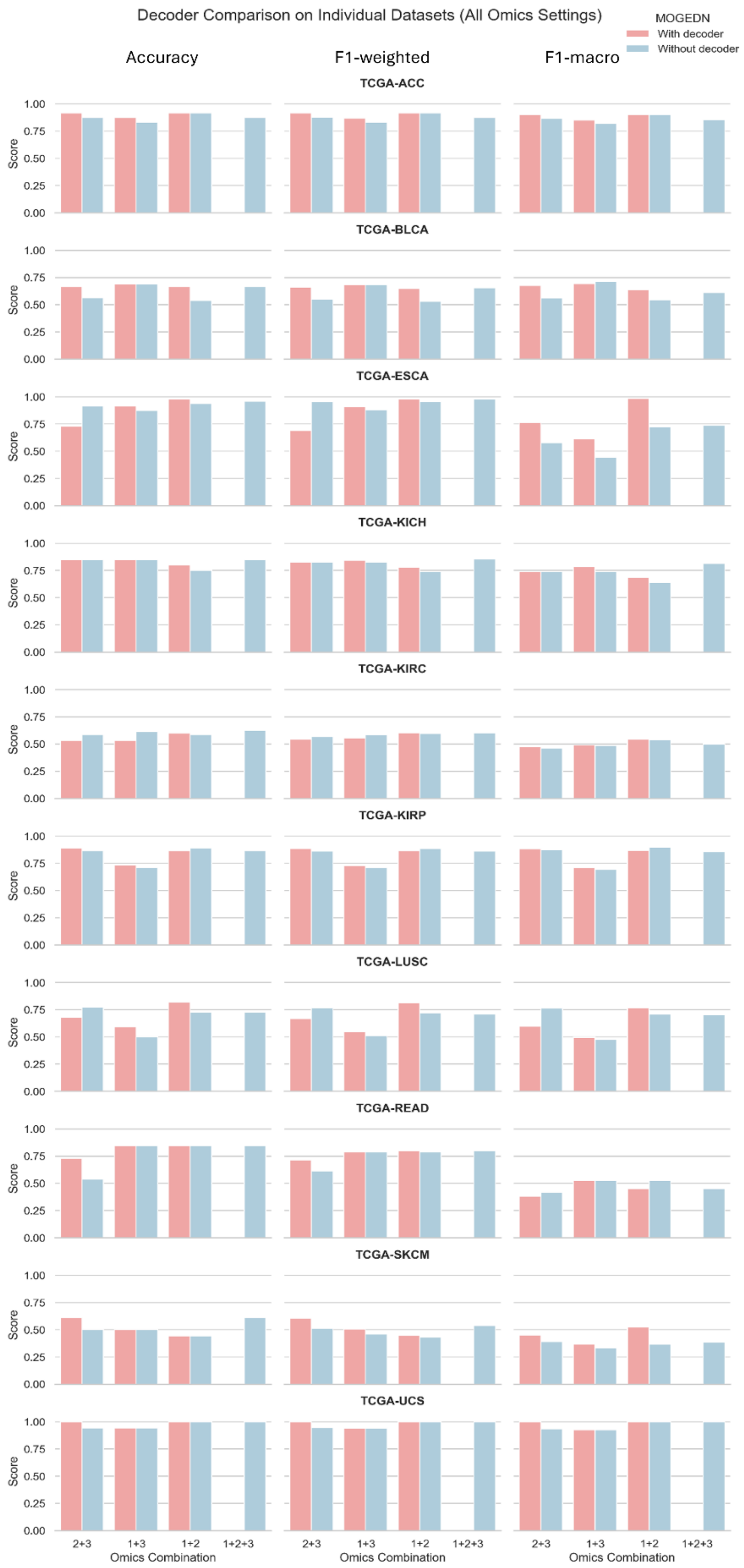

Figure S2 Performance on 10 external TCGA datasets.

Comparison of the proposed model with and without decoder under different missing-omics settings, evaluated per dataset. Omics1: mRNA expression, Omics2: DNA methylation and Omics3: microRNA expression.

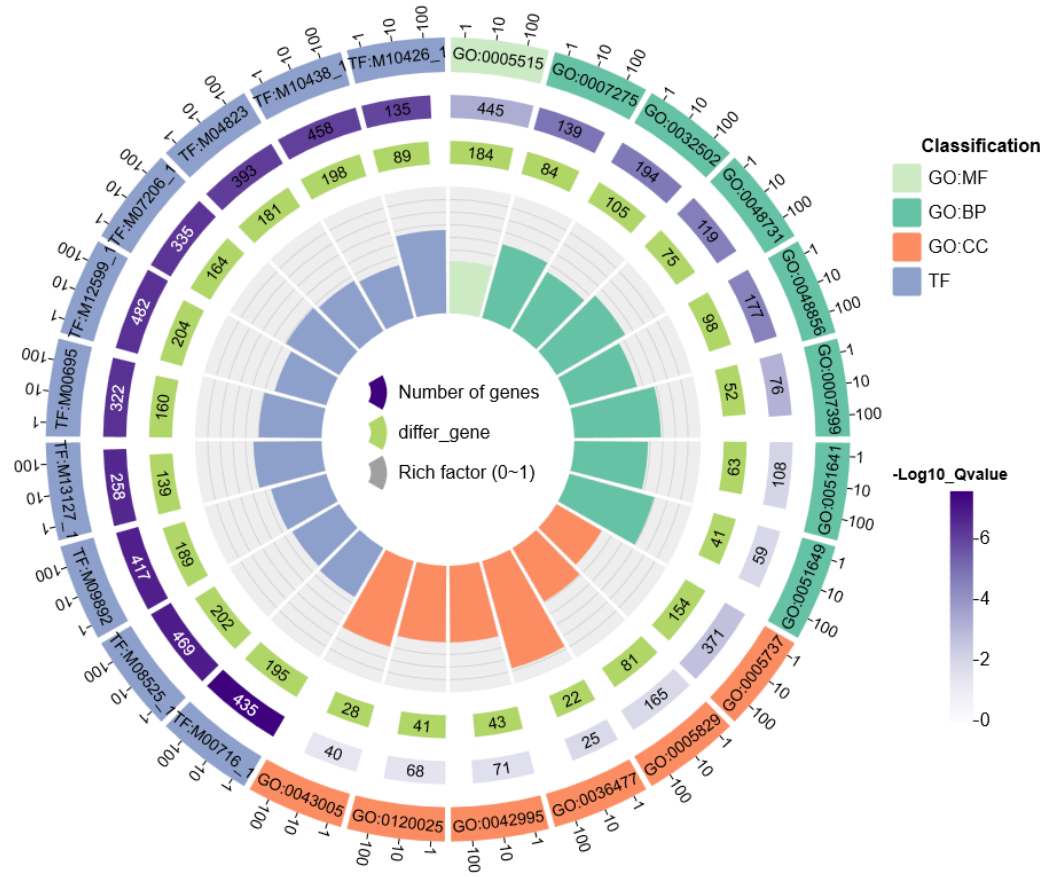

Figure S3 Functional enrichment analysis of DNA methylation-derived genes in TCGA-ACC.

From center to outward: (1) Rich factor (hits / term size) for each term; (2) differ_gene denotes the input set of differentially expressed genes (DEGs) used for enrichment; (3) numbers of gene (hit counts per term). The outer ring lists term IDs (GO/TF). Colors mark annotation categories; the vertical colorbar indicates enrichment score magnitude.

For better readability in this figure, the term size values were scaled.

Algorithm S1 Three-Phase Pretraining Framework

Input: Multi-omics training data $\mathcal{D}_{\text{pretrain}}$, omics list $\mathcal{O} = \{1, 2, 3\}$, mask probability p, reconstruction weight λ

Output: Trained encoder E, decoder D, classifier heads C_o, fusion classifier C

Phase 1: Supervised Encoder and Classifier Pretraining

for *each epoch* **do**
- **for** *each project* $\mathcal{P}$ *in* $\mathcal{D}_{pretrain}$ **do**
  - Encode all omics: $Z_o \leftarrow E_o(X_o)$ for all $o \in \mathcal{O}$
  - Compute classification logits: $y_o \leftarrow C_o(Z_o)$
  - Compute fused prediction: $\hat{y} \leftarrow C(y_1, y_2, y_3)$
  - Compute supervised loss: $\mathcal{L}_{\text{cls}}$
  - Update E_o, C_o, and C to minimize $\mathcal{L}_{\text{cls}}$

Phase 2: Decoder Pretraining (Latent Reconstruction)

Freeze encoder E **for** *each epoch* **do**
- **for** *each project* $\mathcal{P}$ *in* $\mathcal{D}_{pretrain}$ **do**
  - Obtain latent codes: $Z_o \leftarrow E_o(X_o)$
  - **for** *each omic* $o_{mask} \in \mathcal{O}$ **do**
    - Construct decoder input: $Z_{\text{input}} = \text{concat}(Z_j),\ j \neq o_{\text{mask}}$
    - Predict: $\hat{Z}_{o_{\text{mask}}} \leftarrow D(Z_{\text{input}}, o_{\text{mask}})$
    - Compute reconstruction loss: $\mathcal{L}_{\text{recon}} = \text{MSE}(\hat{Z}_{o_{\text{mask}}}, Z_{o_{\text{mask}}})$
    - Update decoder D to minimize $\mathcal{L}_{\text{recon}}$

Phase 3: Joint Pretraining with Decoder Reconstruction

Unfreeze E, freeze D **for** *each epoch* **do**
- **for** *each project* $\mathcal{P}$ *in* $\mathcal{D}_{pretrain}$ **do**
  - Randomly select omic o_{mask} with probability p
  - Replace $Z_{o_{\text{mask}}} \leftarrow D(\text{concat}(Z_j),\ j \neq o_{\text{mask}})$
  - Compute classification loss $\mathcal{L}_{\text{cls}}$
  - Compute total loss: $\mathcal{L} = \mathcal{L}_{\text{cls}} + \lambda \cdot \mathcal{L}_{\text{recon}}$
  - Update E, C_o, and C to minimize $\mathcal{L}$

Algorithm S2 Evaluation Framework

Input: Trained encoder E, decoder D, classifier heads C_o, fusion classifier C, evaluation data $\mathcal{D}_{\text{eval}}$, omics list $\mathcal{O} = \{1, 2, 3\}$

Output: Fine-tuned encoder E, classifier heads C_o, fusion classifier C, performance on evaluation data $\mathcal{D}_{\text{eval}}$

Evaluation on External Dataset

for *each target project* $\mathcal{P}_{eval}$ *in* $\mathcal{D}_{eval}$ **do**

  for *each mask* $o_{mask} \in \{None, 1, 2, 3\}$ **do**

    for *use_decoder* $\in \{True, False\}$ **do**

      Fine-tune E, C_o, and C on $\mathcal{D}_{\text{eval}}^{\text{train}}$ with masked omic

      If use_decoder: use D to reconstruct $Z_{o_{\text{mask}}}$ Evaluate ACC, F1-weighted, F1-macro on $\mathcal{D}_{\text{eval}}^{\text{test}}$

Table S1: Ablation study across different omics combinations for TCGA-ACC

Model	Omics Combination	ACC	F1_weighted	F1_macro
KNN	2+3	0.333	0.265	0.294
LR	2+3	0.708	0.709	0.685
NB	2+3	0.417	0.368	0.387
RF	2+3	0.542	0.540	0.531
Ridge	2+3	0.708	0.709	0.685
SVM	2+3	0.417	0.288	0.319
nn	2+3	0.708	0.697	0.662
MOGONET	2+3	0.875	0.870	0.852
CancerSD	2+3	0.667	0.652	0.652
CLCLSA	2+3	0.625	0.587	0.615
CustOmics	2+3	0.708	0.707	0.684
**MOGEDN-w/oDe**	2+3	0.875	0.878	0.868
**MOGEDN**	2+3	**0.917**	**0.917**	**0.903**
KNN	1+3	0.375	0.282	0.321
LR	1+3	0.500	0.489	0.487
NB	1+3	0.417	0.358	0.369
RF	1+3	0.500	0.510	0.486
Ridge	1+3	0.542	0.544	0.533
SVM	1+3	0.417	0.307	0.352
nn	1+3	0.542	0.543	0.541
MOGONET	1+3	0.458	0.468	0.457
CancerSD	1+3	0.333	0.333	0.333
CLCLSA	1+3	0.667	0.646	0.666
CustOmics	1+3	0.500	0.505	0.484
**MOGEDN-w/oDe**	1+3	0.833	0.834	0.823
**MOGEDN**	1+3	**0.875**	**0.870**	**0.852**
KNN	1+2	0.208	0.089	0.119
LR	1+2	0.542	0.550	0.525
NB	1+2	0.292	0.213	0.245
RF	1+2	0.500	0.481	0.481
Ridge	1+2	0.583	0.591	0.575
SVM	1+2	0.333	0.250	0.278
nn	1+2	0.542	0.560	0.545
MOGONET	1+2	0.667	0.669	0.667
CancerSD	1+2	0.800	0.798	0.794
CLCLSA	1+2	0.583	0.547	0.569
CustOmics	1+2	0.583	0.591	0.566
**MOGEDN-w/oDe**	1+2	**0.917**	**0.917**	**0.903**
**MOGEDN**	1+2	**0.917**	**0.917**	**0.903**
KNN	1+2+3	0.208	0.089	0.119
LR	1+2+3	0.625	0.637	0.615
NB	1+2+3	0.417	0.358	0.369
RF	1+2+3	0.500	0.473	0.474
Ridge	1+2+3	0.708	0.720	0.702
SVM	1+2+3	0.375	0.263	0.291
nn	1+2+3	0.542	0.574	0.548
MOGONET	1+2+3	0.750	0.750	0.717
CancerSD	1+2+3	0.667	0.652	0.652
CLCLSA	1+2+3	0.667	0.643	0.660
CustOmics	1+2+3	0.708	0.708	0.684
**MOGEDN-w/oDe**	1+2+3	**0.875**	**0.876**	**0.856**

Table S2: Ablation study across different omics combinations for TCGA-BLCA

Model	Omics Combination	ACC	F1_weighted	F1_macro
KNN	2+3	0.615	0.580	0.621
LR	2+3	0.615	0.620	0.667
NB	2+3	0.385	0.379	0.300
RF	2+3	0.641	0.614	0.601
Ridge	2+3	**0.692**	**0.702**	**0.699**
SVM	2+3	0.590	0.565	0.450
nn	2+3	0.641	0.645	0.616
MOGONET	2+3	0.487	0.489	0.459
CancerSD	2+3	0.520	0.508	0.500
CLCLSA	2+3	0.667	0.620	0.661
CustOmics	2+3	0.487	0.470	0.468
**MOGEDN-w/oDe**	2+3	0.590	0.583	0.594
**MOGEDN**	2+3	0.641	0.623	0.633
KNN	1+3	0.436	0.306	0.216
LR	1+3	0.590	0.587	0.541
NB	1+3	0.487	0.497	0.494
RF	1+3	0.692	0.687	0.650
Ridge	1+3	0.692	0.697	0.690
SVM	1+3	0.513	0.424	0.315
nn	1+3	0.564	0.577	0.503
MOGONET	1+3	0.641	0.634	0.652
CancerSD	1+3	0.640	0.589	0.507
CLCLSA	1+3	0.615	0.572	0.607
CustOmics	1+3	0.538	0.535	0.501
**MOGEDN-w/oDe**	1+3	0.641	0.639	0.608
**MOGEDN**	1+3	**0.744**	**0.750**	**0.709**
KNN	1+2	0.462	0.393	0.390
LR	1+2	0.667	0.665	0.631
NB	1+2	0.385	0.357	0.383
RF	1+2	0.590	0.581	0.541
Ridge	1+2	0.692	0.693	0.636
SVM	1+2	0.615	0.571	0.445
nn	1+2	0.538	0.547	0.519
MOGONET	1+2	0.538	0.530	0.495
CancerSD	1+2	0.600	0.552	0.641
CLCLSA	1+2	0.667	0.629	0.665
CustOmics	1+2	0.590	0.584	0.560
**MOGEDN-w/oDe**	1+2	0.667	0.657	0.634
**MOGEDN**	1+2	**0.718**	**0.728**	**0.679**
KNN	1+2+3	0.436	0.406	0.406
LR	1+2+3	0.615	0.616	0.587
NB	1+2+3	0.487	0.497	0.494
RF	1+2+3	**0.718**	**0.713**	0.667
Ridge	1+2+3	0.667	0.669	0.600
SVM	1+2+3	0.615	0.589	0.466
nn	1+2+3	0.667	0.664	0.620
MOGONET	1+2+3	0.564	0.568	0.522
CancerSD	1+2+3	0.520	0.507	0.563
CLCLSA	1+2+3	0.692	0.645	0.688
CustOmics	1+2+3	0.564	0.562	0.514
**MOGEDN-w/oDe**	1+2+3	0.692	0.695	**0.688**

Table S3: Ablation study across different omics combinations for TCGA-ESCA

Model	Omics Combination	ACC	F1_weighted	F1_macro
KNN	2+3	0.958	0.948	0.642
LR	2+3	0.958	0.958	0.486
NB	2+3	0.875	0.861	0.578
RF	2+3	0.979	**0.969**	0.658
Ridge	2+3	0.958	0.958	0.486
SVM	2+3	0.958	0.948	0.642
nn	2+3	0.917	0.936	0.379
MOGONET	2+3	0.917	0.927	0.468
CancerSD	2+3	0.935	0.936	0.935
CLCLSA	2+3	**0.979**	0.658	**0.969**
**MOGEDN-w/oDe**	2+3	0.917	0.955	0.578
**MOGEDN**	2+3	0.729	0.691	0.764
KNN	1+3	0.771	0.739	0.485
LR	1+3	0.958	0.958	0.486
NB	1+3	0.688	0.657	0.424
RF	1+3	0.979	**0.969**	0.658
Ridge	1+3	0.958	0.958	0.486
SVM	1+3	0.938	0.927	0.627
nn	1+3	0.875	0.887	0.358
MOGONET	1+3	0.875	0.882	0.443
CancerSD	1+3	0.710	0.811	0.393
CLCLSA	1+3	**0.979**	0.658	**0.969**
**MOGEDN-w/oDe**	1+3	0.875	0.882	0.443
**MOGEDN**	1+3	0.917	0.908	0.614
KNN	1+2	0.896	0.889	0.601
LR	1+2	0.979	0.969	0.658
NB	1+2	0.500	0.496	0.322
RF	1+2	0.958	0.948	0.642
Ridge	1+2	0.979	0.969	0.658
SVM	1+2	0.958	0.948	0.642
nn	1+2	0.917	0.927	0.471
MOGONET	1+2	0.896	0.924	0.373
CancerSD	1+2	0.774	0.774	0.774
CLCLSA	1+2	**0.979**	0.658	0.969
**MOGEDN-w/oDe**	1+2	0.938	0.957	0.724
**MOGEDN**	1+2	0.979	**0.979**	**0.985**
KNN	1+2+3	0.958	0.948	0.642
LR	1+2+3	0.958	0.958	0.486
NB	1+2+3	0.688	0.657	0.424
RF	1+2+3	0.979	0.969	0.658
Ridge	1+2+3	0.958	0.958	0.486
SVM	1+2+3	0.958	0.948	0.642
nn	1+2+3	0.917	0.927	0.628
MOGONET	1+2+3	0.917	0.927	0.468
CancerSD	1+2+3	0.774	0.773	0.765
CLCLSA	1+2+3	**0.979**	0.658	**0.969**
**MOGEDN-w/oDe**	1+2+3	0.958	**0.979**	0.738

Table S4: Ablation study across different omics combinations for TCGA-KICH

Model	Omics Combination	ACC	F1_weighted	F1_macro
KNN	2+3	0.750	0.643	0.429
LR	2+3	0.750	0.643	0.429
NB	2+3	0.850	0.825	0.740
RF	2+3	0.750	0.643	0.429
Ridge	2+3	0.750	0.643	0.429
SVM	2+3	0.750	0.643	0.429
nn	2+3	0.800	0.781	0.688
MOGONET	2+3	0.750	0.708	0.567
CancerSD	2+3	0.615	0.598	0.511
CLCLSA	2+3	0.750	0.429	0.643
CustOmics	2+3	**0.900**	**0.891**	**0.844**
**MOGEDN-w/oDe**	2+3	0.850	0.825	0.740
**MOGEDN**	2+3	0.850	0.825	0.740
KNN	1+3	0.750	0.643	0.429
LR	1+3	0.750	0.643	0.429
NB	1+3	0.750	0.643	0.429
RF	1+3	0.750	0.643	0.429
Ridge	1+3	0.750	0.643	0.429
SVM	1+3	0.750	0.643	0.429
nn	1+3	0.650	0.673	0.627
MOGONET	1+3	0.600	0.626	0.560
CancerSD	1+3	0.615	0.625	0.575
CLCLSA	1+3	0.750	0.429	0.643
CustOmics	1+3	0.500	0.533	0.451
**MOGEDN-w/oDe**	1+3	**0.850**	0.825	0.740
**MOGEDN**	1+3	**0.850**	**0.844**	**0.785**
KNN	1+2	0.700	0.618	0.412
LR	1+2	0.750	0.643	0.429
NB	1+2	0.750	0.643	0.429
RF	1+2	0.750	0.643	0.429
Ridge	1+2	**0.800**	0.745	0.608
SVM	1+2	0.750	0.643	0.429
nn	1+2	0.750	0.708	0.567
MOGONET	1+2	0.500	0.533	0.451
CancerSD	1+2	0.538	0.538	0.458
CLCLSA	1+2	0.750	0.429	0.643
CustOmics	1+2	0.450	0.483	0.373
**MOGEDN-w/oDe**	1+2	0.750	0.740	0.642
**MOGEDN**	1+2	**0.800**	**0.781**	**0.688**
KNN	1+2+3	0.700	0.618	0.412
LR	1+2+3	0.750	0.643	0.429
NB	1+2+3	0.750	0.643	0.429
RF	1+2+3	0.750	0.643	0.429
Ridge	1+2+3	0.750	0.643	0.429
SVM	1+2+3	0.750	0.643	0.429
nn	1+2+3	0.650	0.636	0.498
MOGONET	1+2+3	0.750	0.740	0.642
CancerSD	1+2+3	0.615	0.625	0.575
CLCLSA	1+2+3	0.750	0.429	0.643
CustOmics	1+2+3	0.750	0.708	0.567
**MOGEDN-w/oDe**	1+2+3	**0.850**	**0.854**	**0.812**

Table S5: Ablation study across different omics combinations for TCGA-KIRC

Model	Omics Combination	ACC	F1_weighted	F1_macro
KNN	2+3	0.467	0.409	0.302
LR	2+3	0.573	0.546	0.442
NB	2+3	0.427	0.411	0.331
RF	2+3	0.507	0.445	0.324
Ridge	2+3	0.480	0.468	0.381
SVM	2+3	0.440	0.343	0.237
nn	2+3	0.467	0.460	0.365
MOGONET	2+3	0.427	0.421	0.344
CancerSD	2+3	0.314	0.290	0.204
CLCLSA	2+3	0.507	0.380	**0.476**
CustOmics	2+3	0.480	0.445	0.342
**MOGEDN-w/oDe**	2+3	**0.587**	**0.567**	0.461
**MOGEDN**	2+3	0.533	0.542	0.476
KNN	1+3	0.373	0.257	0.162
LR	1+3	0.560	0.536	0.427
NB	1+3	0.360	0.325	0.227
RF	1+3	0.467	0.426	0.307
Ridge	1+3	0.440	0.421	0.310
SVM	1+3	0.360	0.210	0.113
nn	1+3	0.480	0.489	0.379
MOGONET	1+3	0.493	0.506	0.410
CancerSD	1+3	0.294	0.282	0.234
CLCLSA	1+3	0.480	0.316	0.433
CustOmics	1+3	0.480	0.458	0.350
**MOGEDN-w/oDe**	1+3	**0.613**	**0.586**	0.485
**MOGEDN**	1+3	0.533	0.558	**0.490**
KNN	1+2	0.427	0.347	0.233
LR	1+2	0.533	0.501	0.374
NB	1+2	0.400	0.394	0.308
RF	1+2	0.467	0.394	0.273
Ridge	1+2	0.533	0.498	0.374
SVM	1+2	0.453	0.363	0.256
nn	1+2	0.533	0.519	0.417
MOGONET	1+2	0.467	0.494	0.450
CancerSD	1+2	0.421	0.421	0.336
CLCLSA	1+2	0.453	0.285	0.402
CustOmics	1+2	0.533	0.510	0.428
**MOGEDN-w/oDe**	1+2	0.587	0.599	0.540
**MOGEDN**	1+2	**0.600**	**0.605**	**0.543**
KNN	1+2+3	0.493	0.409	0.289
LR	1+2+3	0.547	0.511	0.397
NB	1+2+3	0.347	0.312	0.217
RF	1+2+3	0.507	0.428	0.304
Ridge	1+2+3	0.520	0.490	0.374
SVM	1+2+3	0.427	0.320	0.220
nn	1+2+3	0.480	0.483	0.387
MOGONET	1+2+3	0.493	0.507	0.422
CancerSD	1+2+3	0.431	0.421	0.336
CLCLSA	1+2+3	0.507	0.370	0.472
CustOmics	1+2+3	0.573	0.532	0.405
**MOGEDN-w/oDe**	1+2+3	**0.627**	**0.602**	**0.501**

Table S6: Ablation study across different omics combinations for TCGA-KIRP

Model	Omics Combination	ACC	F1_weighted	F1_macro
KNN	2+3	0.533	0.410	0.269
LR	2+3	0.711	0.668	0.568
NB	2+3	0.467	0.386	0.247
RF	2+3	0.578	0.482	0.351
Ridge	2+3	0.733	0.705	0.628
SVM	2+3	0.556	0.455	0.296
nn	2+3	0.756	0.748	0.666
MOGONET	2+3	0.733	0.724	0.689
CancerSD	2+3	0.700	0.692	0.483
CLCLSA	2+3	0.600	0.371	0.517
CustOmics	2+3	0.756	0.728	0.660
**MOGEDN-w/oDe**	2+3	0.867	0.864	0.873
**MOGEDN**	2+3	**0.889**	**0.888**	**0.882**
KNN	1+3	0.467	0.297	0.159
LR	1+3	0.644	0.599	0.508
NB	1+3	0.444	0.301	0.161
RF	1+3	0.511	0.401	0.262
Ridge	1+3	0.711	0.688	0.619
SVM	1+3	0.467	0.333	0.197
nn	1+3	0.533	0.526	0.408
MOGONET	1+3	0.444	0.466	0.429
CancerSD	1+3	0.700	0.710	0.658
CLCLSA	1+3	0.578	0.346	0.485
CustOmics	1+3	0.689	0.658	0.609
**MOGEDN-w/oDe**	1+3	0.711	0.710	0.693
**MOGEDN**	1+3	**0.733**	**0.729**	**0.710**
KNN	1+2	0.467	0.306	0.164
LR	1+2	0.600	0.528	0.420
NB	1+2	0.467	0.302	0.162
RF	1+2	0.578	0.482	0.351
Ridge	1+2	0.600	0.539	0.442
SVM	1+2	0.511	0.398	0.254
nn	1+2	0.711	0.698	0.626
MOGONET	1+2	0.578	0.581	0.530
CancerSD	1+2	0.733	0.704	0.519
CLCLSA	1+2	0.556	0.290	0.448
CustOmics	1+2	0.644	0.603	0.512
**MOGEDN-w/oDe**	1+2	**0.889**	**0.888**	**0.897**
**MOGEDN**	1+2	0.867	0.866	0.871
KNN	1+2+3	0.467	0.306	0.164
LR	1+2+3	0.667	0.624	0.532
NB	1+2+3	0.444	0.301	0.161
RF	1+2+3	0.556	0.437	0.292
Ridge	1+2+3	0.689	0.659	0.575
SVM	1+2+3	0.511	0.407	0.268
nn	1+2+3	0.733	0.734	0.682
MOGONET	1+2+3	0.800	0.797	0.785
CancerSD	1+2+3	0.733	0.742	0.644
CLCLSA	1+2+3	0.556	0.286	0.441
CustOmics	1+2+3	0.711	0.654	0.515
**MOGEDN-w/oDe**	1+2+3	**0.867**	**0.860**	**0.858**

Table S7: Ablation study across different omics combinations for TCGA-LUSC

Model	Omics Combination	ACC	F1_weighted	F1_macro
KNN	2+3	0.500	0.400	0.321
LR	2+3	0.591	0.570	0.521
NB	2+3	0.364	0.301	0.230
RF	2+3	0.591	0.492	0.421
Ridge	2+3	0.545	0.506	0.448
SVM	2+3	0.500	0.375	0.299
nn	2+3	0.636	0.606	0.542
MOGONET	2+3	0.636	0.586	0.503
CancerSD	2+3	0.643	0.589	0.548
CLCLSA	2+3	0.318	0.186	0.260
CustOmics	2+3	0.636	0.617	0.563
**MOGEDN-w/oDe**	2+3	**0.773**	**0.768**	**0.764**
**MOGEDN**	2+3	0.682	0.669	0.597
KNN	1+3	0.409	0.263	0.161
LR	1+3	0.500	0.375	0.291
NB	1+3	0.409	0.323	0.257
RF	1+3	0.455	0.335	0.236
Ridge	1+3	0.500	0.388	0.299
SVM	1+3	0.455	0.324	0.229
nn	1+3	0.500	0.471	0.431
MOGONET	1+3	0.409	0.307	0.188
CancerSD	1+3	0.500	0.477	0.476
CLCLSA	1+3	0.364	0.282	0.344
CustOmics	1+3	0.182	0.116	0.107
**MOGEDN-w/oDe**	1+3	0.500	0.512	0.476
**MOGEDN**	1+3	**0.591**	**0.549**	**0.494**
KNN	1+2	0.455	0.319	0.227
LR	1+2	0.591	0.492	0.430
NB	1+2	0.409	0.323	0.257
RF	1+2	0.636	0.558	0.497
Ridge	1+2	0.636	0.577	0.537
SVM	1+2	0.364	0.242	0.148
nn	1+2	0.636	0.627	0.622
MOGONET	1+2	0.636	0.620	0.596
CancerSD	1+2	0.643	0.577	0.591
CLCLSA	1+2	0.409	0.378	0.404
CustOmics	1+2	0.182	0.127	0.113
**MOGEDN-w/oDe**	1+2	0.727	0.720	0.708
**MOGEDN**	1+2	**0.818**	**0.815**	**0.769**
KNN	1+2+3	0.455	0.328	0.232
LR	1+2+3	0.591	0.511	0.438
NB	1+2+3	0.409	0.323	0.257
RF	1+2+3	0.636	0.577	0.537
Ridge	1+2+3	0.636	0.597	0.546
SVM	1+2+3	0.409	0.298	0.210
nn	1+2+3	0.591	0.548	0.470
MOGONET	1+2+3	0.636	0.609	0.535
CancerSD	1+2+3	0.714	0.636	0.632
CLCLSA	1+2+3	0.455	0.309	0.377
CustOmics	1+2+3	0.636	0.617	0.563
**MOGEDN-w/oDe**	1+2+3	**0.727**	**0.711**	**0.702**

Table S8: Ablation study across different omics combinations for TCGA-READ

Model	Omics Combination	ACC	F1_weighted	F1_macro
KNN	2+3	0.808	**0.737**	0.304
LR	2+3	0.808	0.722	0.298
NB	2+3	0.808	0.722	0.298
RF	2+3	0.808	0.722	0.298
Ridge	2+3	0.808	0.722	0.298
SVM	2+3	0.808	0.722	0.298
nn	2+3	0.538	0.626	0.269
MOGONET	2+3	0.731	0.714	0.221
CancerSD	2+3	0.706	0.730	0.276
CLCLSA	2+3	**0.808**	0.298	**0.722**
CustOmics	2+3	0.654	0.687	0.212
**MOGEDN-w/oDe**	2+3	0.538	0.613	0.417
**MOGEDN**	2+3	0.731	0.714	0.381
KNN	1+3	0.808	0.722	0.298
LR	1+3	0.808	0.722	0.298
NB	1+3	0.808	0.722	0.298
RF	1+3	0.808	0.722	0.298
Ridge	1+3	0.808	0.722	0.298
SVM	1+3	0.808	0.722	0.298
nn	1+3	0.500	0.584	0.222
MOGONET	1+3	0.500	0.580	0.280
CancerSD	1+3	0.706	0.770	0.415
CLCLSA	1+3	0.808	0.298	**0.722**
CustOmics	1+3	0.500	0.586	0.226
**MOGEDN-w/oDe**	1+3	**0.846**	**0.789**	0.527
**MOGEDN**	1+3	**0.846**	**0.789**	0.527
KNN	1+2	0.808	0.722	0.298
LR	1+2	0.808	0.722	0.298
NB	1+2	0.808	0.722	0.298
RF	1+2	0.808	0.722	0.298
Ridge	1+2	0.808	0.722	0.298
SVM	1+2	0.808	0.722	0.298
nn	1+2	0.423	0.538	0.167
MOGONET	1+2	0.385	0.462	0.191
CancerSD	1+2	0.765	0.765	0.289
CLCLSA	1+2	0.808	0.298	**0.722**
CustOmics	1+2	0.269	0.321	0.166
**MOGEDN-w/oDe**	1+2	**0.846**	0.789	0.527
**MOGEDN**	1+2	**0.846**	**0.802**	0.452
KNN	1+2+3	0.808	0.722	0.298
LR	1+2+3	0.808	0.722	0.298
NB	1+2+3	0.808	0.722	0.298
RF	1+2+3	0.808	0.722	0.298
Ridge	1+2+3	0.808	0.722	0.298
SVM	1+2+3	0.808	0.722	0.298
nn	1+2+3	0.462	0.570	0.176
MOGONET	1+2+3	0.769	0.718	0.296
CancerSD	1+2+3	0.765	0.765	0.289
CLCLSA	1+2+3	0.808	0.298	**0.722**
CustOmics	1+2+3	0.731	0.714	0.221
**MOGEDN-w/oDe**	1+2+3	**0.846**	**0.802**	0.452

Table S9: Ablation study across different omics combinations for TCGA-SKCM

Model	Omics Combination	ACC	F1_weighted	F1_macro
KNN	2+3	0.500	0.389	0.233
LR	2+3	**0.611**	0.530	0.357
NB	2+3	0.444	0.308	0.123
RF	2+3	0.556	0.420	0.272
Ridge	2+3	0.556	0.486	0.325
SVM	2+3	0.500	0.333	0.133
nn	2+3	0.389	0.437	0.227
MOGONET	2+3	0.444	0.444	0.267
CancerSD	2+3	0.462	0.402	0.394
CLCLSA	2+3	0.556	0.272	0.420
CustOmics	2+3	0.333	0.319	0.206
**MOGEDN-w/oDe**	2+3	0.500	0.511	0.390
**MOGEDN**	2+3	**0.611**	**0.605**	**0.450**
KNN	1+3	0.389	0.299	0.189
LR	1+3	0.444	0.308	0.123
NB	1+3	0.500	0.333	0.133
RF	1+3	0.556	0.429	0.238
Ridge	1+3	**0.611**	0.526	0.419
SVM	1+3	0.500	0.333	0.133
nn	1+3	0.556	**0.541**	**0.522**
MOGONET	1+3	0.389	0.402	0.283
CancerSD	1+3	0.462	0.369	0.213
CLCLSA	1+3	0.500	0.133	0.333
CustOmics	1+3	0.167	0.189	0.120
**MOGEDN-w/oDe**	1+3	0.500	0.463	0.333
**MOGEDN**	1+3	0.500	0.505	0.369
KNN	1+2	0.500	0.385	0.206
LR	1+2	0.556	0.420	0.272
NB	1+2	0.500	0.333	0.133
RF	1+2	0.500	0.346	0.138
Ridge	1+2	0.556	**0.470**	0.319
SVM	1+2	0.500	0.333	0.133
nn	1+2	0.333	0.326	0.175
MOGONET	1+2	0.278	0.265	0.228
CancerSD	1+2	0.462	0.365	0.225
CLCLSA	1+2	**0.556**	0.272	0.420
CustOmics	1+2	0.167	0.121	0.090
**MOGEDN-w/oDe**	1+2	0.444	0.430	0.366
**MOGEDN**	1+2	0.444	0.448	**0.526**
KNN	1+2+3	0.389	0.304	0.166
LR	1+2+3	0.556	0.420	0.272
NB	1+2+3	0.500	0.333	0.133
RF	1+2+3	0.556	0.420	0.272
Ridge	1+2+3	**0.667**	**0.564**	0.417
SVM	1+2+3	0.500	0.333	0.133
nn	1+2+3	0.278	0.293	0.202
MOGONET	1+2+3	0.444	0.416	0.298
CancerSD	1+2+3	0.538	0.410	0.267
CLCLSA	1+2+3	0.556	0.272	**0.420**
CustOmics	1+2+3	0.333	0.304	0.233
**MOGEDN-w/oDe**	1+2+3	0.611	0.538	0.386

Table S10: Ablation study across different omics combinations for TCGA-UCS

Model	Omics Combination	ACC	F1_weighted	F1_macro
KNN	2+3	0.778	0.719	0.600
LR	2+3	0.944	0.942	0.926
NB	2+3	0.611	0.631	0.579
RF	2+3	0.944	0.942	0.926
Ridge	2+3	**1.000**	**1.000**	**1.000**
SVM	2+3	0.722	0.606	0.419
nn	2+3	0.944	0.946	0.935
MOGONET	2+3	0.778	0.789	0.766
CancerSD	2+3	0.636	0.636	0.542
CLCLSA	2+3	0.778	0.723	0.778
CustOmics	2+3	0.667	0.679	0.662
**MOGEDN-w/oDe**	2+3	0.944	0.946	0.935
**MOGEDN**	2+3	**1.000**	**1.000**	**1.000**
KNN	1+3	0.778	0.719	0.600
LR	1+3	0.889	0.879	0.839
NB	1+3	0.778	0.758	0.679
RF	1+3	**0.944**	**0.942**	**0.926**
Ridge	1+3	0.889	0.879	0.839
SVM	1+3	0.722	0.606	0.419
nn	1+3	0.611	0.630	0.600
MOGONET	1+3	0.722	0.736	0.699
CancerSD	1+3	0.818	0.783	0.694
CLCLSA	1+3	0.833	0.734	0.806
CustOmics	1+3	0.444	0.444	0.444
**MOGEDN-w/oDe**	1+3	**0.944**	**0.942**	**0.926**
**MOGEDN**	1+3	**0.944**	**0.942**	**0.926**
KNN	1+2	0.833	0.806	0.734
LR	1+2	0.944	0.942	0.926
NB	1+2	0.722	0.606	0.419
RF	1+2	0.889	0.879	0.839
Ridge	1+2	0.944	0.942	0.926
SVM	1+2	0.722	0.606	0.419
nn	1+2	0.889	0.894	0.875
MOGONET	1+2	0.889	0.894	0.875
CancerSD	1+2	0.909	0.913	0.895
CLCLSA	1+2	0.889	0.839	0.879
CustOmics	1+2	0.778	0.789	0.766
**MOGEDN-w/oDe**	1+2	**1.000**	**1.000**	**1.000**
**MOGEDN**	1+2	**1.000**	**1.000**	**1.000**
KNN	1+2+3	0.778	0.719	0.600
LR	1+2+3	0.944	0.942	0.926
NB	1+2+3	0.778	0.758	0.679
RF	1+2+3	0.833	0.806	0.734
Ridge	1+2+3	**1.000**	**1.000**	**1.000**
SVM	1+2+3	0.722	0.606	0.419
nn	1+2+3	0.944	0.946	0.935
MOGONET	1+2+3	0.778	0.789	0.766
CancerSD	1+2+3	0.909	0.913	0.895
CLCLSA	1+2+3	0.889	0.839	0.879
CustOmics	1+2+3	0.778	0.789	0.766
**MOGEDN-w/oDe**	1+2+3	**1.000**	**1.000**	**1.000**

Table S11: Results across different omics combinations for the largest dataset TCGA-BRCA

Model	Omics Combination	ACC	F1_weighted	F1_macro
MOGONET	2+3	0.716	0.737	0.614
CancerSD	2+3	0.575	0.568	0.463
CLCLSA	2+3	**0.838**	0.613	**0.814**
CustOmics	2+3	0.817	**0.804**	0.671
**MOGEDN-w/oDe**	2+3	0.769	0.782	0.680
**MOGEDN**	2+3	**0.821**	**0.829**	**0.744**
MOGONET	1+3	0.734	0.755	0.562
CancerSD	1+3	0.706	0.682	0.571
CLCLSA	1+3	**0.830**	0.569	**0.802**
CustOmics	1+3	0.790	0.789	0.623
**MOGEDN-w/oDe**	1+3	0.777	**0.789**	0.684
**MOGEDN**	1+3	**0.795**	**0.803**	**0.705**
MOGONET	1+2	0.738	0.754	0.636
CancerSD	1+2	0.569	0.585	0.488
CLCLSA	1+2	**0.808**	0.495	**0.768**
CustOmics	1+2	**0.821**	**0.815**	0.676
**MOGEDN-w/oDe**	1+2	0.734	0.749	0.670
**MOGEDN**	1+2	0.747	**0.768**	**0.691**
MOGONET	1+2+3	0.769	0.780	0.682
CancerSD	1+2+3	0.706	0.682	0.571
CLCLSA	1+2+3	**0.838**	0.637	**0.820**
CustOmics	1+2+3	0.808	**0.810**	0.694
**MOGEDN-w/oDe**	1+2+3	**0.817**	**0.827**	**0.721**

Table S12: TCGA-KICH associated biomarkers from multi-omics data

Omics type	Biomarkers
mRNA expression (20)	DLL3, MYH1, **SLC17A2**, DPPA5P4, ACTRT1, TAS2R3, FUT5, PADI3, ALPG, KLK11, PATE1, FAM170B, TOPAZ1, PMS2P6, CDV3P1, LINC00469, CDKN2AIPNLP1, CALML3, LINC00305, EDDM3B
DNA methylation (20)	**PSMD10**, ATG4A, CAPZA1, ST7L, **CASZ1**, FMN2, MAGOH, LHX8, WDR65, EBNA1BP2, MTA3, LRRFIP1, TMBIM1, PNKD, GRHL1, ABCG5, COQ10B, FAM98A, SNED1, UMPS
miRNA expression (10)	hsa-mir-6882, hsa-mir-4726, hsa-mir-3619, hsa-mir-5706, hsa-mir-4522, hsa-mir-6855, hsa-mir-5187, hsa-mir-4741, hsa-mir-664b, **hsa-mir-148b**

Note: Bold biomarkers have peer-reviewed evidence linking them to kidney renal cancers(KICH).

Table S13: TCGA-KIRC associated biomarkers from multi-omics data

Omics type	Biomarkers
mRNA expression (20)	SPP2, LINC02566, ADGB, BTG4, MBL2, CACNG2, CCDC13–AS1, RAX2, DRD5P2, CIMAP1A, DEFB119, RNY4P24, RNU6–704P, RNU6–188P, RNU6–304P, RNU1–100P, RNU1–55P, OR2H1, RNU6–1279P, RNU6–409P
DNA methylation (20)	ARX, DDX26B, DLG3, CCDC21, FLJ37453, GDAP2, WDR3, KIAA0040, **ALPL**, PUSL1, ACAP3, IGSF9, CDK18, C1orf35, AHCYL1, ASAP3, FBLIM1, STX6, RWDD3, **CDC20**
miRNA expression (10)	hsa–mir–658, **hsa–mir–190b**, **hsa–mir–148b**, hsa–mir–6870, hsa–mir–6793, **hsa–mir–139**, hsa–mir–3620, hsa–mir–3941, hsa–mir–6731, hsa–mir–6756

Note: Bold biomarkers have peer-reviewed evidence linking them to kidney renal clear cell carcinoma (KIRC).

Table S14: TCGA-KIRP associated biomarkers from multi-omics data

Omics type	Biomarkers
mRNA expression (20)	NT5C1A, DPPA5P4, PRLHR, SEMG1, SLX1A, SSTR4, SCN1A, SPATA16, **FABP7**, HSFY1, TENT5D, NUDT4B, OR2T29, OR7E87P, C14orf180, OR11H13P, LINC02692, MUC2, MIR302D, RNA5SP370
DNA methylation (20)	CAMK1G, PPM1J, FOXD2, HIPK1, HFE2, PPP1R1C, WDSUB1, TUBA4A, TUBA4B, GIGYF2, ALG3, ECE2, PARP3, RRP9, VGLL3, MLF1IP, AFF1, WHSC2, **ARHGAP26**, CXXC5
miRNA expression (10)	hsa-mir-3611, hsa-mir-4724, hsa-mir-6894, hsa-mir-6891, **hsa-mir-324**, hsa-mir-4452, hsa-mir-548s, **hsa-mir-25**, hsa-mir-7113, hsa-mir-3614

Note: Bold biomarkers have peer-reviewed evidence linking them to papillary renal cell carcinoma (KIRP) or renal cell carcinoma (RCC) in general.

Table S15: TCGA-LUSC associated biomarkers from multi-omics data

Omics type	Biomarkers
mRNA expression (20)	PPP1R2C, PPY, PSG8, TSPAN16, LYZL2, OR52W1, CDKN2AIPNLP1, OR3A1, WFDC10A, EDDM3B, **GRIN2A**, COX8C, FGF16, RN7SKP184, RNU6–1216P, RNU6–1301P, RNA5SP378, RNA5SP161, RNA5SP469, RNU4–51P
DNA methylation (20)	UBA1, ADORA3, HPDL, DR1, MMACHC, CCDC163P, HIPK1, STIL, USP40, **EPCAM**, SEPT2, HDLBP, LOC100287216, SH3RF3, RNASEH1, MYT1L, PLB1, CCDC12, C3orf31, **ALCAM**
miRNA expression (10)	hsa–mir–6839, hsa–mir–3178, hsa–mir–4522, **hsa–mir–1246**, hsa–mir–378d–1, **hsa–mir–135b**, hsa–mir–548ai, hsa–mir–651, hsa–mir–4658, hsa–mir–6726

Note: Bold biomarkers have peer-reviewed evidence linking them to liver hepatocellular carcinoma (LUSC) or nonsmall-cell lung cancer.

Table S16: TCGA-READ associated biomarkers from multi-omics data

**Omics type**	**Biomarkers**
mRNA expression (20)	OLFM3, ARL4AP1, NKX2-4, ENAM, CRP, RNF17, **IRS4**, GUCA1C, GOLGA6D, GABRA6, ACTL7B, FEZF2, CYP4Z2P, CRYAA, KNCN, UMOD, KRT72, RLN3, SHANK2-AS3, OR1A1
DNA methylation (20)	RPGR, C1orf35, LOC149134, **LEPR**, LEPROT, PAX7, C1orf9, MTF2, TMEM214, TMEM44, STAC, **SEC62**, DGKG, **IL12A**, CDKN2AIP, PDE6B, NBLA00301, HAND2, NPR3, COL23A1
miRNA expression (10)	hsa-mir-3617, hsa-mir-6882, hsa-mir-6856, **hsa-mir-98**, hsa-mir-1255a, hsa-mir-6783, hsa-mir-6839, hsa-mir-6827, **hsa-mir-320a**, hsa-mir-4753

Note: Biomarkers in **bold** are backed by peer-reviewed evidence linking them to rectum adenocarcinoma or colorectal cancer (READ).

Table S17: TCGA-SKCM associated biomarkers from multi-omics data

**Omics type**	**Biomarkers**
mRNA expression (20)	SERPINA4, DBX1, TSHB, SLC17A4, TFF1, MYL1, OR6F1, MT1B, OR4K17, HORMAD2, KRTAP13-2, ACTL7A, LINC00207, OR11H13P, ANKRD20A19P, OR2B4P, OR7E13P, RNU4-80P, RNU5B-1, RNA5SP382
DNA methylation (20)	PAGE4, DLG3, **AHCYL1**, MFSD4, PTBP2, **C1QC**, TMEM63A, AHDC1, TBX15, NPHP4, CCDC85A, MARCH7, ACTR2, KIF5C, DYNC2LI1, IFT122, MBD4, USP13, SCN5A, OTUD4
miRNA expression (10)	**hsa-mir-548ai**, hsa-mir-767, **hsa-mir-382**, hsa-mir-3117, hsa-mir-6087, hsa-mir-3920, hsa-mir-1256, hsa-mir-548q, hsa-mir-607, **hsa-mir-204**

Note: Biomarkers in **bold** have peer-reviewed evidence linking them to skin cutaneous melanoma (SKCM).

Table S18: TCGA–UCS associated biomarkers from multi-omics data

**Omics type**	**Biomarkers**
mRNA expression (20)	BPIFA3, RHOXF2, FSHB, ASB15, G6PC2, GPR101, OR9K2, FNDC9, OR3A4P, OR4Q3, OR52E8, OR6C70, OR13F1, KRTAP19–5, DUSP21, BLOC1S6P1, **MIR96**, RNA5SP174, RNU6–642P, RNA5SP325
DNA methylation (20)	HMGN5, **S1PR1**, PSRC1, NIPAL3, PTPRU, MMEL1, CDC42SE1, MLLT11, ZNF142, BCS1L, SH3RF3, C3orf24, HPS3, ADAD1, SORCS2, MLF1IP, FAF2, CCNJL, C5orf32, MDN1
miRNA expression (10)	hsa–mir–6858, hsa–mir–4777, hsa–mir–4467, hsa–mir–6895, **hsa–mir–30d**, hsa–mir–3692, hsa–mir–7845, hsa–mir–548ao, hsa–mir–4665, hsa–mir–1537

Note: Entries in **bold** have peer-reviewed evidence linking them to uterine carcinosarcoma or, when specific data are scarce, to uterine/endometrial malignancies in general.
